# Comparison of degradation behavior and osseointegration of 3D powder-printed calcium magnesium phosphate cement scaffolds with alkaline or acid post-treatment

**DOI:** 10.3389/fbioe.2022.998254

**Published:** 2022-09-28

**Authors:** Katharina Kowalewicz, Anja-Christina Waselau, Franziska Feichtner, Anna-Maria Schmitt, Manuel Brückner, Elke Vorndran, Andrea Meyer-Lindenberg

**Affiliations:** ^1^ Clinic for Small Animal Surgery and Reproduction, Ludwig-Maximilians-University of Munich, Munich, Germany; ^2^ Department for Functional Materials in Medicine and Dentistry, University of Würzburg, Würzburg, Germany

**Keywords:** stanfieldite, farringtonite, newberyite, 3D powder printing, scaffold, biocompatibility, osseointegration, degradable bone substitute

## Abstract

Due to the positive effects of magnesium substitution on the mechanical properties and the degradation rate of the clinically well-established calcium phosphate cements (CPCs), calcium magnesium phosphate cements (CMPCs) are increasingly being researched as bone substitutes. A post-treatment alters the materials’ physical properties and chemical composition, reinforcing the structure and modifying the degradation rate. By alkaline post-treatment with diammonium hydrogen phosphate (DAHP, (NH_4_)_2_HPO_4_), the precipitation product struvite is formed, while post-treatment with an acidic phosphate solution [e.g., phosphoric acid (PA, H_3_PO_4_)] results in precipitation of newberyite and brushite. However, little research has yet been conducted on newberyite as a bone substitute and PA post-treatment of CMPCs has not been described in the accessible literature so far. Therefore, in the present study, the influence of an alkaline (DAHP) or acid (PA) post-treatment on the biocompatibility, degradation behavior, and osseointegration of cylindrical scaffolds (*h* = 5.1 mm, *Ø* = 4.2 mm) produced from the ceramic cement powder Ca_0.75_Mg_2.25_(PO_4_)_2_ by the advantageous manufacturing technique of three-dimensional (3D) powder printing was investigated *in vivo*. Scaffolds of the material groups Mg225d (DAHP post-treatment) and Mg225p (PA post-treatment) were implanted into the cancellous part of the lateral femoral condyles in rabbits. They were evaluated up to 24 weeks by regular clinical, X-ray, micro-computed tomographic (µCT), and histological examinations as well as scanning electron microscopy (SEM) and energy dispersive X-ray spectroscopy (EDX) analysis and compared with tricalcium phosphate (TCP). All materials showed excellent biocompatibility and rapid osseointegration. While TCP degraded only slightly, the CMPCs showed almost complete degradation. Mg225d demonstrated significantly faster loss of form and demarcability from surrounding bone, scaffold volume reduction, and significantly greater degradation on the side towards the bone marrow than to the cortex than Mg225p. Simultaneously, numerous bone trabeculae have grown into the implantation site. While these were mostly located on the side towards the cortex in Mg225d, they were more evenly distributed in Mg225p and showed almost the same structural characteristics as physiological bone after 24 weeks in Mg225p. Based on these results, the acid post-treated 3D powder-printed Mg225p is a promising degradable bone substitute that should be further investigated.

## 1 Introduction

Bone substitutes are needed for the reconstruction of large bone defects that occur for example due to trauma, tumors, infections or congenital defects (Kheirallah and Almeshaly, 2016). Due to a longer life expectancy, there is a sharp increase in musculoskeletal diseases such as osteoporosis, bone infections or metastases and fractures, leading to an increase in bone-related medical treatments (Agarwal and García, 2015). Autografts are still the gold standard in the surgical management of critical size bone defects (Kolk et al., 2012). However, natural bone substitutes do not apply to all types of bone defects and carry various risks such as donor site infections or additional trauma in autografts and allografts (Laurie et al., 1984; Arrington et al., 1996) as well as rejection of the implanted material or transmission of diseases through allografts and xenografts (Keating and McQueen, 2001; Campana et al., 2014). Due to these disadvantages, there has been constant research into the development of synthetic bone substitutes in recent years, which have the advantages of defined porosity and chemical composition, precision-fit for any bone defect, sterility and unlimited availability (Zimmermann and Moghaddam, 2011; Kolk et al., 2012; Kheirallah and Almeshaly, 2016). Synthetic bone substitutes exist in various application forms such as granules, scaffolds, blocks or injectable pastes (Fillingham and Jacobs, 2016; Kheirallah and Almeshaly, 2016).

For the production of three-dimensional (3D), dimensionally stable bone substitutes, various additive manufacturing techniques are currently being investigated (Castilho et al., 2014a; Roseti et al., 2017). Using medical imaging data [e.g., computed tomography (CT)] or computer-aided design (CAD) models, the precision-fit production of patient specific implants is possible, which is particularly advantageous in case of large and anatomically complex bone defects (Peters et al., 2006; Castilho et al., 2014a; Castilho et al., 2014b; Brunello et al., 2016; Roseti et al., 2017; Zhang et al., 2019). 3D powder printing has emerged as a promising additive manufacturing technique with great potential for the layer-by-layer production of individual synthetic bone substitutes (Vorndran et al., 2008; Castilho et al., 2014a; Roseti et al., 2017). Raw materials are for example calcium phosphate (CaP), magnesium phosphate (MgP) or calcium magnesium phosphate (CaMgP) cement powders (Klammert et al., 2010b; Castilho et al., 2014b; Kowalewicz et al., 2021). Due to the high printing accuracy of 3D powder printing, dimensionally stable objects with specifically adjustable macro- and microstructure can be optimally produced, allowing the best possible adjustment of the scaffold properties to the tissue type to be replaced and therefore causing an optimal cell reaction *in vivo* (Castilho et al., 2014b; Brunello et al., 2016; Zhang et al., 2019). 3D powder-printed scaffolds also have a high microporosity (up to more than 30 vol%) (Castilho et al., 2014b), which promotes nutrient diffusion, vascularization of the scaffold and cell ingrowth (Boyan et al., 1996; Karageorgiou and Kaplan, 2005).

The ideal synthetic bone graft substitute is biocompatible, has similar mechanical properties as bone and undergoes physicochemical as well as cellular degradation while being replaced by newly formed bone (Moore et al., 2001; Kolk et al., 2012). Due to the similarity of their chemical composition to the mineral phase of bone, an excellent biocompatibility as well as osteoconductive and in some cases even osteoinductive properties, CaPs represent the currently favored synthetic bone substitutes (Dorozhkin and Epple, 2002; Gross and Berndt, 2002; LeGeros, 2002; Habibovic et al., 2008; LeGeros, 2008; Bohner et al., 2020). The majority of CaP bioceramics are chemically based on hydroxyapatite (HA), both types of tricalcium phosphate (α-TCP, ß-TCP) and/or their multiphase formulations (Dorozhkin, 2013). Calcium phosphate cements (CPCs) have been commercially available for years and are clinically applied as solids and cement pastes (Dorozhkin, 2008; Rentsch et al., 2012; Lodoso-Torrecilla et al., 2021). However, many CPCs, like most CaPs, have the disadvantage of incomplete degradation over months to years due to a low solubility under physiological conditions (Kurashina et al., 1997; Frakenburg et al., 1998; Bohner et al., 2003; Ambard and Mueninghoff, 2006; Kanter et al., 2014). The wider clinical application of these cements is also limited by their mechanical properties, as CPCs are brittle, have low impact resistance, and variable compressive strengths (Ambard and Mueninghoff, 2006; Dorozhkin, 2008). Magnesium phosphate cements (MPCs) have a higher compressive strength and, due to their higher solubility, exhibit a greater chemical degradation potential than CPCs, which is associated with faster *in-vivo* resorption and higher bone ingrowth, qualifying them as a suitable alternative bone substitute (Mestres and Ginebra, 2011; Ostrowski et al., 2016; Nabiyouni et al., 2018; Haque and Chen, 2020; Gefel et al., 2022). However, unlike CPCs, the biomedical application of MPCs has hardly been investigated so far. It is therefore reasonable to combine the MPCs with their superior biological properties with the well-established CPCs (Ostrowski et al., 2016; Nabiyouni et al., 2018). Several studies have shown that calcium magnesium phosphate cements (CMPCs) exhibit improved biological properties as bone substitutes than the single components and an excellent biocompatibility, good and increasing osseointegration, fast degradation, and rapid replacement by newly formed trabecular bone have been described (Wu et al., 2008a; Wu et al., 2008b; Klammert et al., 2010a; Jia et al., 2010; Wei et al., 2010; Vorndran et al., 2011; Zeng et al., 2012; Ewald et al., 2019; Fuchs et al., 2021).

In a previous short-term *in-vivo* study by Kowalewicz et al. (2021), CMPC scaffolds fabricated with the advantageous production method of 3D powder printing have yet been investigated and especially the alkaline (diammonium hydrogen phosphate, DAHP) post-treated material Mg225d showed promising initial results regarding degradation and osseointegration. Besides post-treatment with DAHP, which results in precipitation of struvite and has yet also been investigated *in vivo* for CMPC pastes or granules by other authors (Ewald et al., 2019; Fuchs et al., 2021), the post-treatment can also be carried out with an acidic phosphate solution (Klammert et al., 2010a; Klammert et al., 2011; Gefel et al., 2022), resulting in precipitation of newberyite and brushite. However, newberyite has been little researched as bone substitute so far and there is no accessible literature available on the post-treatment of CMPCs with phosphoric acid (PA). Therefore, the aim of the present study was the development, characterization, and first *in-vivo* long-term investigation of 3D powder-printed CMPC scaffolds with different physical and chemical properties due to alkaline (DAHP) or acid (PA) post-treatment. The two CMPC material groups Mg225d (DAHP post-treatment) and Mg225p (PA post-treatment) were compared with each other. TCP scaffolds served as reference. For 6, 12, and 24 weeks, respectively, the influence of the different post-treatments on biocompatibility, degradation, and osseointegration behavior of the cylindrical scaffolds was evaluated in a non-weight-bearing borehole defect in the cancellous part of the lateral femoral condyles in rabbits.

## 2 Materials and methods

### 2.1 Production and characterization of the scaffolds

#### 2.1.1 Production of the scaffolds

**FIGURE 1:**
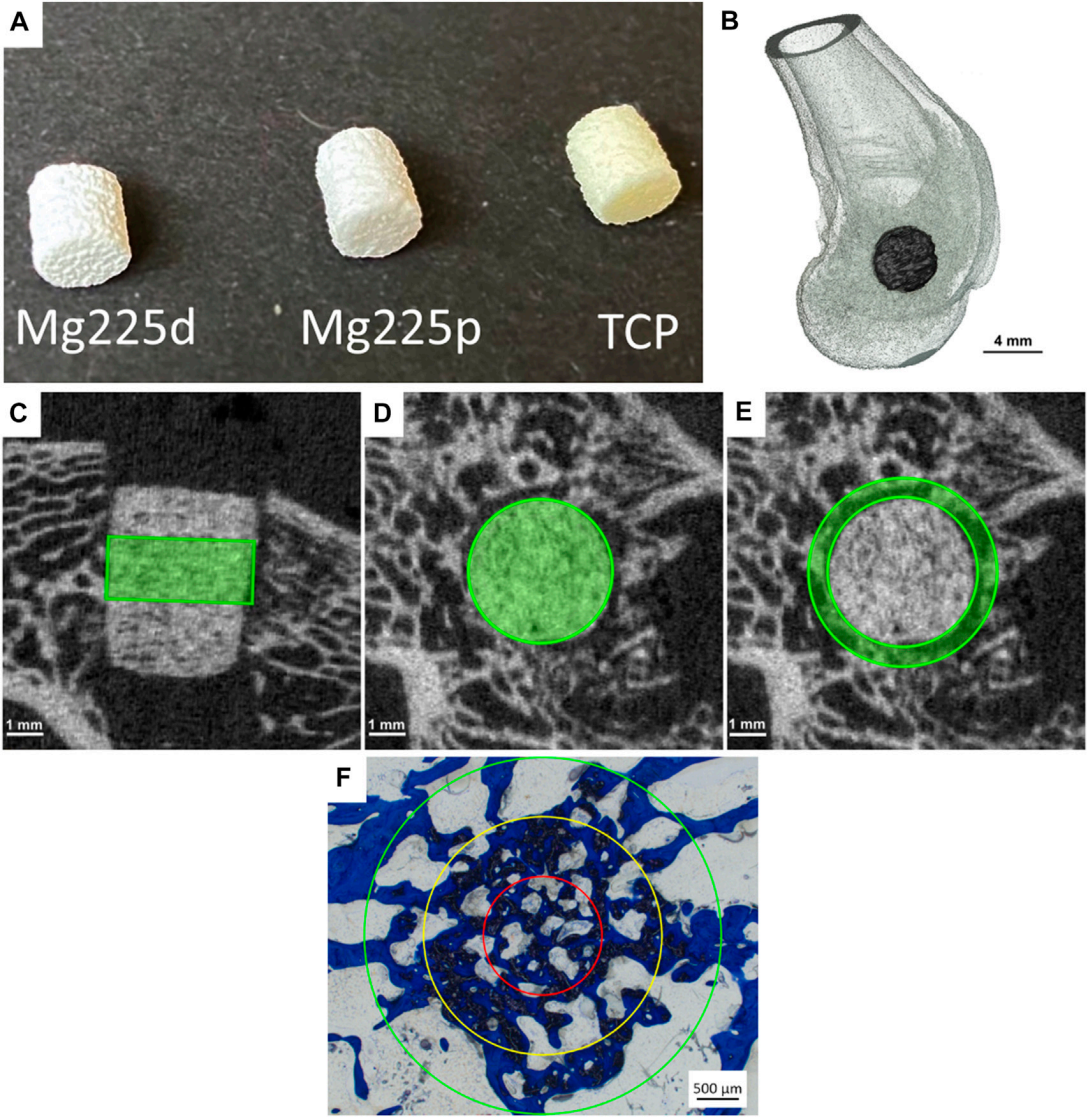
**(A)** Mg225d, Mg225p and TCP scaffolds prior to implantation (*h* = 5.1 mm, *Ø* = 4.2 mm). **(B)** Three-dimensional (3D) reconstruction of the distal rabbit femur with implanted scaffold in the lateral condyle in lateral view. **(C)** Cylindrical region of interest (ROI) in the scaffold center for measurement of scaffold volume (SV) and scaffold surface/scaffold volume (SS/SV) in the original *in-vivo* µCT scan (*h* = 60 slices ≙1.82 mm) and **(D)** in the reoriented *in-vivo* µCT scan (*Ø* = 140 voxels (≙4.24 mm)). **(E)** Second hollow cylindrical ROI for measurement of bone volume (BV), trabecular number (Tb.N), trabecular thickness (Tb.Th) and trabecular separation (Tb.Sp) in the scaffold environment in the reoriented *in-vivo* µCT scan (inner ring: *Ø* = 144 voxels (≙4.36 mm), outer ring: *Ø* = 180 voxels (≙5.45 mm), *h* = 60 slices ≙1.82 mm). **(F)** ROIs for the semi-quantitative histological examination: red = inner ring (IR), yellow = medial ring (MR), green = outer ring (OR). *Ø* (OR) = 4.24 mm.

Raw material for the production of the cylindrical CMPC scaffolds (*h* = 5.1 mm, *Ø* = 4.2 mm) was the ceramic cement powder Ca_0.75_Mg_2.25_(PO_4_)_2_. Reference implants with the same dimensions were made of tricalcium phosphate (TCP, Ca_3_(PO_4_)_2_) cement (Figure 1A). Therefore, mixtures of calcium hydrogen phosphate (CaHPO_4_, J.T. Baker, Philippsburg, United States), calcium carbonate (CaCO_3_, Merck KGaA, Darmstadt, Germany), magnesium hydrogen phosphate (MgHPO_4_∙3H_2_O, Alfa Aesar, Kandel, Germany), and magnesium hydroxide (Mg(OH)_2_, VWR International GmbH, Darmstadt, Germany) were prepared in specific molar ratios (Table 1). These powder mixtures were sintered, ground to powders with a particle size <355 µm using a ball mill, and mixed with 4 wt% hydroxypropyl methylcellulose (HPMC) before processing in powder printing (Kowalewicz et al., 2021). The scaffolds were fabricated using a 3D powder printer (ZP310, ZCorp., Burlington, United States) (Kowalewicz et al., 2021). After depowdering of the scaffolds with compressed air, the scaffolds underwent a heat treatment to compact the ceramic and burn out the HPMC, which included a debinding step at 500°C for 2 h and a phase-dependent final sintering temperature. Ca_0.75_Mg_2.25_(PO_4_)_2_ scaffolds were sintered at a final temperature of 1,100°C for 4 h, while TCP scaffolds were sintered at 1,350°C for 4 h. After this sintering process, a second sintering of TCP was performed at 1,000°C for 4 h in order to increase the conversion rate of α-TCP into ß-TCP.

**TABLE 1:** Powder synthesis: Composition of the cement powders and sintering temperature of the raw powders.

Cement powder	Raw material (mol)				Sintering temperature (°C)
	CaHPO_4_	CaCO_3_	MgHPO_4_ ∙ 3H_2_O	Mg(OH)_2_	
Ca_0.75_Mg_2.25_(PO_4_)_2_	0.50	0.25	1.50	0.75	1,100
Ca_3_(PO_4_)_2_	2.00	1.00	—	—	1,350

The CMPC scaffolds were then divided into two material groups, which were subjected to different post-treatments. Both post-treatment variants were carried out at room temperature. One half of the scaffolds (Mg225d) received post-treatment by immersion (24 h) in an aqueous diammonium hydrogen phosphate solution (3.5 M DAHP, (NH_4_)_2_HPO_4_, Merck KGaA, Darmstadt, Germany), the other half (Mg225p) was completely infiltrated four times with a phosphoric acid solution (2.0 M PA, (H_3_PO_4_, Merck KGaA, Darmstadt, Germany). The infiltrations with PA were done with a sufficient amount of liquid (100–160 μl PA per scaffold) to completely fill the pores and the scaffolds were dried at room temperature for 24 h after each infiltration. The post-treated scaffolds were washed in distilled water for 1 h and in phosphate-buffered saline (PBS) (Sigma-Aldrich, Taufkirchen, Germany) for 10 min. For this purpose, the scaffolds were placed in a Petri dish, completely covered with the washing medium and placed on a rocking table for uniform mixing of the medium.

Before implantation, all scaffolds were γ-sterilized by BBF Sterilisationsservice GmbH (Kernen, Germany) with a radiation of >25 kGy.

#### 2.1.2 Physical and chemical properties of the scaffolds

The compressive strength was measured using a static universal testing machine (Z010, Zwick GmbH, Ulm, Germany). For this purpose, the post-treated scaffolds (*n* = 14 per material), which had been stored in PBS for 1 h, were measured in a wet condition with a 10 kN load cell, at a pre-load of 1 N and a test speed of 1 mm/min.

Open porosity and pore size distribution were determined on three scaffolds per material type using a mercury porosimeter (Pascal 140/440, Thermo Fisher Scientific Inc., Waltham, MA, United States). For each measurement, an entire cylindrical scaffold was used. Porosity was measured in a pressure range from 0.01 kPa to 400 MPa and data was analyzed using the software SOLID (SOLver of Intrusion Data Ver. 1.6.5, Thermo Fisher Scientific Inc. Waltham, MA, United States).

The chemical composition of the post-treated and sterile scaffolds was determined by X-ray powder diffraction and Rietveld analysis. For qualitative phase composition, three scaffolds per material type were ground into powder and each powder was analyzed individually using the Bruker Corporations D8 Advance X-ray diffractometer (Bruker Corporations, Karlsruhe, Germany) with monochromatic radiation (*λ* = 1.541 Å). The measurement was performed in the scan type locked coupled, within a 2θ-angular range of 10–40° with an increment of 0.02°, a measurement speed of 0.5 s/step, and under rotation of the measurement cuvette of 15 rpm. The following reference files from the JCPDS database were used for analysis: ß-Ca_3_(PO_4_)_2_ (ß-tricalcium phosphate, PDF Ref. 09-0169), α-Ca_3_(PO_4_)_2_ (α-tricalcium phosphate, PDF Ref. 09-0348), Mg_3_(PO_4_)_2_ (farringtonite; PDF Ref. 33-0876), MgHPO_4_·3H_2_O (newberyite, PDF Ref. 35-0780), Ca_4_Mg_5_(PO_4_)_6_ (stanfieldite; PDF Ref. 11-0231), and NH_4_MgPO_4_·6H_2_O (struvite, PDF Ref. 15-0762). Using the Rietveld method (Rietveld, 1969; Reid and Hendry, 2006), a quantitative phase analysis was carried out. Bruker Corporations’ TOPAS V6 software (Bruker Corporations, Billerica, MA, United States) was used to perform this analysis.

#### 2.1.3 Scanning electron microscopy and energy dispersive X-ray spectroscopy analysis of the scaffolds before implantation

For SEM (scanning electron microscopy) and EDX (energy dispersive X-ray spectroscopy) analysis, the scaffolds were fixed in 4% buffered formalin solution (Roth, Karlsruhe, Germany), dehydrated in an ascending series of alcohol (Roth, Karlsruhe, Germany) and defatted with xylene (Roth, Karlsruhe, Germany). Embedding was performed with a resin embedding system based on methyl methacrylate (Technovit^®^ 9100, Heraeus Kulzer, Wehrheim, Germany) according to the manufacturer’s instructions. After polymerization, thin sections (thickness = 4 μm, *n* = 1 per material) were prepared using an automatic rotary microtome (RM2255 Leica, Wetzlar, Germany). After transfer to a water bath, the thin sections were mounted on glass slides (Glaswarenfabrik Karl Hecht, Sondheim, Germany) coated with ponal-poly-L-lysine. They were then stretched with 96% ethanol, covered with a polyethylene film (Heraeus Kulzer, Hanau, Germany) and dried in a slide press at 37°C for 2 days. Prior to SEM with a field emission electron microscope (Crossbeam CB 340, Zeiss, Oberkochen, Germany), the thin sections were coated with platinum (thickness = 4 nm) using a sputter coater (Leica EM ACE600, Leica Mikrosysteme GmbH, Wetzlar, Germany). For EDX imaging, a system with silicon drift detector (INCA Energy 350 AzTec Advanced system with silicon drift detector) from Oxford Instruments, Abingdon, United Kingdom was used. Using an accelerating voltage of 5–10 keV, the scaffolds were examined at a magnification of ×24 and ×100. SEM was used to assess the surface texture and pore structure of the scaffolds, while EDX was used to analyze the distribution of chemical phases based on the occurrence of magnesium (Mg), calcium (Ca) and phosphorus (P).

### 2.2 Animal model

The animal experiment was approved by the competent authority (Government of Upper Bavaria) according to paragraph 8 of the German Animal Welfare Act (approval number ROB 55.2-2532.Vet_02-19-64). For this study, 36 female adult Zika rabbits (Asamhof, Kissing, Germany) weighing 4.26 ± 0.27 kg were divided into three groups with an observation period of 6, 12 and 24 weeks post-surgery, respectively. According to a fixed implantation scheme, eight scaffolds per material (Mg225d, Mg225p, TCP) and time group were implanted and examined.

The implantation of the scaffolds into the lateral femoral condyles of both hind limbs was performed following a previous study by Kowalewicz et al. (2021). The animals received enrofloxacin (10 mg/kg, Orniflox^®^, CP-Pharma GmbH, Burgdorf, Germany) as antibiotic and meloxicam (0.3 mg/kg, Melosus^®^, Albrecht GmbH, Aulendorf, Germany) as analgesic per os prior to surgery. Induction of anesthesia was performed by intramuscular application of ketamine (15 mg/kg, Aneskin^®^, Albrecht GmbH, Aulendorf, Germany) and medetomidine (0.25 mg/kg, Dorbene vet^®^, Zoetis Deutschland GmbH, Berlin, Germany). The airways were secured by endotracheal intubation and the animals were placed in supine position after shaving and aseptic preparation of the surgical area. Anesthesia was maintained by inhalation of isoflurane (1.5–2 vol%, simultaneously supply of oxygen 1 L/min). During surgery, the rabbits received 10 μg/kg/h fentanyl intravenously (Fentadon^®^, CP-Pharma GmbH, Burgdorf, Germany) as pain medication.

Surgical access was performed through a skin incision in the area of the right lateral femoral condyle. After dissection of the muscles and visualization of the condyle using a raspatory, an approximately 5 mm deep hole was drilled in the cancellous part of the condyle directly above the attachment of the lateral collateral ligament. The cylindrical scaffold was inserted accurately into the borehole (Figure 1B). Wound closure of the soft tissue (Monosyn 4/0, B. Braun SE, Melsungen, Germany) and the skin (Optilene 4/0, B. Braun SE, Melsungen, Germany) was performed. After completion of the surgical procedure on the first side, the animals received intravenous buprenorphine (20 μg/kg, Bupresol^®^, CP-Pharma GmbH, Burgdorf, Germany) for pain management. The contralateral femur was operated using the same surgical procedure. Immediately after surgery, an *in-vivo* micro-computed tomographic (µCT) examination (see Section 2.4) was performed of both hindlimbs, and radiographs (see Section 2.3) were obtained in two views. Finally, medetomidine was antagonized by intramuscular application of atipamezole (25 mg/kg, Atipam^®^, Albrecht GmbH, Aulendorf, Germany).

During the first 14 days after surgery, the animals were clinically and orthopedically examined daily, especially with regard to lameness and pain, and a daily wound assessment was performed. For 5 days, the animals received enrofloxacin (10 mg/kg, Orniflox^®^, CP-Pharma GmbH, Burgdorf, Germany) as antibiotic and meloxicam (0.3 mg/kg, Melosus^®^, Albrecht GmbH, Aulendorf, Germany) for pain management per os once daily. At fixed time points (6, 12, and 24 weeks after surgery, respectively), euthanasia of the animals was carried out in accordance with animal welfare regulations by intravenous application of propofol (5 mg/kg, Narcofol^®^, CP-Pharma GmbH, Burgdorf, Germany) and pentobarbital (200–230 mg/kg, Narkodorm^®^, CP-Pharma GmbH, Burgdorf, Germany). The femora were collected and adherent soft tissue was removed. The scaffold-bone-complexes were extracted using a diamond band saw (cut-grinder, Walter Messner GmbH, Oststeinbek, Germany).

### 2.3 X-ray examination

Immediately after surgery and at predefined time points (every 2 weeks until week 12, hereinafter every 4 weeks until week 24), a radiological examination of the rabbits’ hind limbs was performed in two views [ventrodorsal (VD), mediolateral (ML)]. The examinations were conducted with the settings 54.9 kV and 4.5 mA (Multix Select DR, Siemens GmbH, Erlangen, Germany). Using the software dicomPACS^®^ vet (Ver.8.9.5, Oehm und Rehbein GmbH, Rostock, Germany), the visibility of the scaffolds in the different X-ray views was assessed by two observers.

### 2.4 *In-vivo* µCT examination

Immediately after surgery and at the same predefined time points as the radiological examinations, the lateral femoral condyles of the rabbits were examined in an *in-vivo* µCT (Xtreme CT II, Scanco Medical, Zurich, Switzerland). The scans were performed with the settings 30.3 µm isotropic voxel size, 68 kV voltage, 1,000/180° projections, and 200 ms integration time. For this purpose, the rabbits were placed in supine position with stretched hindlimbs. For the scan immediately after surgery, anesthesia was maintained with isoflurane (0.8–1.0 vol%, simultaneously oxygen supply 1.5–2 L/min). For the subsequent scans, anesthesia was induced as for surgery. Due to the shorter duration of anesthesia, the animals were not intubated for these scans but received oxygen (1.5–2 L/min) via a laryngeal mask (v-gel^®^ rabbit, Docsinnovent Ltd., London, United Kingdom).

#### 2.4.1 Semi-quantitative evaluation of *in-vivo* µCT scans

With a special modified scoring system (Kowalewicz et al., 2021), the following parameters were assessed by two observers: Scaffold demarcability based on gray value and structure, degradation properties, loss of cylindrical form, occurrence and distinctivity of a resorption zone (area within the scaffold volume characterized by a markedly lower gray value than the scaffold material), and scaffold-bone-contact. Score values from 0–2 were assigned for each parameter examined (Table 2). To obtain the cross-sectional view of the scaffolds with surrounding cancellous bone, it was necessary to rotate the original scan using the software µCT Evaluation Program V6.6 (Scanco Medical, Zurich, Switzerland).

**TABLE 2:** Scoring system for the semi-quantitative evaluation of the *in-vivo* µCT scans.

Parameter	Score 0	Score 1	Score 2
Scaffold demarcability (based on grey value and density/structure)	Scaffold not demarcable from surrounding bone tissue	Scaffold partially demarcable from surrounding bone tissue	Scaffold completely demarcable from surrounding bone tissue
Degradation properties	Scaffold uniformly degraded	Scaffold half close to the bone marrow more degraded	Scaffold half close to the bone marrow no longer visible
Loss of form	Cylindrical form no longer recognizable	Cylindrical form partially recognizable	Cylindrical form clearly recognizable
Distinctivity of a resorption zone (area within the scaffold volume with a markedly darker gray value than the scaffold material)	No resorption zone distinctive	Resorption zone indistinctly delineated	Resorption zone distinctly delineated
Scaffold-bone-contact	Broad contact area between scaffold and bone, numerous bone trabeculae on scaffold, no gap visible	Multiple bone trabeculae between scaffold and surrounding bone, barely visible gap	No contact between scaffold and surrounding bone, clear gap between bone and scaffold

#### 2.4.2 Quantitative evaluation of *in-vivo* µCT scans

To calculate various degradation and osseointegration parameters, it was necessary to define material-specific thresholds. The following thresholds (Th) were established by assessing the grey values of the different scaffolds in the scans directly after surgery (*n* = 6 per material): Mg225d: 140, Mg225p: 149, TCP: 219. For cancellous bone at the same location, the Th 142 was determined using µCT scans of both lateral femoral condyles of adult Zika rabbit cadavers (*n* = 4) with intact femurs. Scaffold volume (SV) and scaffold surface area to volume ratio (SS/SV) were calculated following the studies of Kowalewicz et al. (2021), Augustin et al. (2020), and Kleer et al. (2019) in a region of interest (ROI) in the scaffold center. This ROI included a cylinder with a diameter of 140 voxels (≙4.24 mm) and a height of 60 slices (≙1.82 mm) (Figures 1C,D). Bone volume (BV), trabecular number (Tb.N), trabecular thickness (Tb.Th), and trabecular separation (Tb.Sp) in the scaffold environment were calculated also based on the studies of Kowalewicz et al. (2021), Augustin et al. (2020) and Kleer et al. (2019) in a second hollow cylindrical ROI (inner ring: *Ø* = 144 voxel (≙4.36 mm), outer ring: *Ø* = 180 voxel (≙5.45 mm), height = 60 slices) (Figure 1E). To establish reference values for cancellous bone, the cancellous part of both femoral condyles of cadavers of adult Zika rabbits (*n* = 4) with intact femora was examined. All calculations were performed using the software µCT Evaluation Program V6.6 (Scanco Medical, Zurich, Switzerland).

### 2.5 µCT 80 examination

After euthanasia of the animals and extraction of the scaffold-bone-complexes, these were processed as described previously in Section 2.1.3. After polymerization, the sample blocks were scanned using a µCT 80 (Scanco Medical, Zurich, Switzerland). The scans were performed with settings of 10 µm isotropic voxel size, 70 kV voltage and 600 ms integration time.

#### 2.5.1 Semi-quantitative evaluation of µCT 80 scans

Using a scoring system developed for this study, the occurrence and location of trabecular structures in cross and longitudinal section of the scaffold volume were assessed in the complete scan by two observers. Score values from 0–2 were assigned for both parameters examined (Table 3).

**TABLE 3:** Scoring system for the semi-quantitative evaluation of the µCT 80 scans.

Parameter	Score 0	Score 1	Score 2
Trabecular structures in the scaffold volume: Cross-sectional view	Numerous trabecular structures visible up to the center of the scaffold radius	Trabecular structures visible in >50% of the scaffold radius, not extending to the center	Trabecular structures visible in the outer <50% of the scaffold radius
Trabecular structures in the scaffold volume: Longitudinal view	Trabecular structures throughout the scaffold volume	Amount of trabecular structures close to the cortex markedly larger than close to the bone marrow	Trabeculae mainly located close to the cortex, few trabeculae close to the bone marrow

#### 2.5.2 Quantitative evaluation of µCT 80 scans

Trabecular number (Tb.N), trabecular thickness (Tb.Th), and trabecular separation (Tb.Sp) were measured within a defined cylindrical ROI in the scaffold center [*h* = 182 voxels (≙1.82 mm), *Ø* = 424 voxels (≙4.24 mm)]. Eight scans per material were used to determine the Ths for cancellous bone (144–235). Reference values for cancellous bone at the same location were determined using scans of eight lateral femoral condyles from adult Zika rabbit cadavers with intact femora. All calculations were performed using the software µCT Evaluation Program V6.6 (Scanco Medical, Zurich, Switzerland).

### 2.6 Histological examination

Thick sections (thickness = 40 µm) of the embedded scaffold-bone-complexes were produced according to the cutting-grinding technique of Donath and Breuner (1982) using a diamond band saw (cut-grinder, Walter Messner GmbH, Oststeinbek, Germany) and a grinding machine (lap-grinder, Walter Messner GmbH, Oststeinbek, Germany). A central section of each implanted scaffold was routinely stained with toluidine blue (0.1% toluidine blue O solution, Waldeck, Münster, Germany) (Willbold and Witte, 2010; Huehnerschulte et al., 2012). The longitudinal axis of the cylinder was perpendicular to the cutting surface. The µCT 80 scans were used to determine the scaffold position within the condyle.

#### 2.6.1 Semi-quantitative evaluation of histological sections

The stained histological sections were assessed by two observers using a microscope (Zeiss Axio Imager Z.2, Carl Zeiss Microscopy GmbH, Jena, Germany). Following von Doernberg et al. (2006), the implantation area was divided into three ROIs at ×25 magnification using three rings (IR = inner ring, MR = medial ring, OR = outer ring), with the diameter of the OR (*Ø* = 4.24 mm) corresponding to the scaffold diameter (Figure 1F). In each ROI, a scoring system modified according to von Doernberg et al. (2006), Kleer-Reiter et al. (2021) and Augustin et al. (2022) was used to assess in the initial scaffold cross section the percentage area of scaffold material, bone, granulation tissue/bone marrow and resorption zone (cell- and connective tissue-rich annular zone within the scaffold cross section) as well as the percentage of scaffold material enclosed by bone (Table 4). In each ROI, a second scoring system (Table 4) was used to evaluate the ingrowing tissue at cellular level (fibrous cells/tissue, adipocytes, precursor cells, vascularization/blood vessels, macrophages, foreign body cells (FBC), osteoblasts, osteoclasts, osteoid) in a field of view with fixed position at ×100 magnification. Score values from 0–3 were assigned for each parameter examined (Table 4).

**TABLE 4:** Scoring system for the semi-quantitative histological evaluation of tissue and cells.

Parameter	Score 0	Score 1	Score 2	Score 3
Tissue level				
Scaffold material	0%	1%–25%	26%–50%	>50%
Scaffold material enclosed by bone	0%	1%–25%	26%–50%	>50%
New bone (thin trabeculae, dark blue colored)	0%	1%–25%	26%–50%	>50%
Remodeled bone (trabecular thickness as in environment, light blue colored)	0%	1%–25%	26%–50%	>50%
Granulation tissue/bone marrow	0%	1%–25%	26%–50%	>50%
Cell-rich resorption zone (fibrous tissue)	0%	1%–25%	26%–50%	>50%
Cell level				
Tissue ingrowth				
Fibrous cells/tissue	None/physiological for bone marrow	Slightly increased	Medium increased	Highly increased
Adipocytes	None	Few	Moderate	Many
Precursor cells (bone marrow activity)	None	Few	Moderate	Many
Vascularization^a^ (blood vessels)	None	Few	Moderate	Many
Foreign body reaction				
Macrophages^b^	None	Few	Moderate	Many
Foreign body cells^b^	None	Few	Moderate	Many
Bone tissue and cells				
Osteoblasts^b^	None	Few	Moderate	Many
Osteoclasts^b^	None	Few	Moderate	Many
Osteoid	None	Sporadic	Thin layer	Thick layer

a None = 0, Few = 1–3, Moderate = 4–6, Many = 7+ blood vessels.

b None = 0, Few = 1–5, Moderate = 6–10, Many = 10+ cells.

#### 2.6.2 Quantitative evaluation (histomorphometry)

In addition to the semi-quantitative analysis, the thick sections from the scaffold center were quantitatively analyzed by histomorphometry. For this purpose, images of the cross sections (Zeiss Axio Imager 2, Carl Zeiss Microscopy GmbH, Jena, Germany) were taken at ×20 magnification using the Zeiss Axio Cam Mrc digital camera and the software Zeiss ZEN 3.0 (Carl Zeiss Microscopy GmbH, Jena, Germany). The images were evaluated using the software Zeiss ZEN 3.0 (Carl Zeiss Microscopy GmbH, Jena, Germany). The percentage area of scaffold material, ingrown bone tissue, and soft tissue (granulation tissue, bone marrow) was measured within a predefined circle (*Ø* = 1,060 pixels ≙ diameter of the OR of the semiquantitative examination (*Ø* = 4.24 mm) ≙ scaffold diameter), which was placed centrally around the initial implantation area.

### 2.7 SEM and EDX analysis of the unstained histological sections

Histological thin sections (*n* = 2 per material and time group) were prepared and examined by SEM and EDX analysis as previously described in Section 2.1.3. The scaffold center was examined with a magnification of ×28 and ×500. In SEM, the osseointegration of the scaffolds was assessed morphologically based on the surface texture of the thin sections, while in EDX, the presence of material particles was determined based on the occurrence of magnesium ions (CMPCs).

### 2.8 Statistics

Statistical analysis of the compressive strength and porosity of the scaffolds was performed by analysis of variance (ANOVA) followed by Tukey’s post hoc test using Origin (OriginPro 2022, OriginLab, Northampton, MA, United States). The collected *in-vivo* data were analyzed with SPSS Statistics 26 (IBM Company, Armonk, United States). Using the Shapiro–Wilk test, data were tested for normal distribution. Normally distributed data were analyzed using analysis of variance (ANOVA followed by Tukey post hoc test/Welch-ANOVA followed by Games–Howell post-hoc test). For non-normally distributed data, testing for significant differences was done using Kruskal–Wallis test with one-way analysis of variance (ANOVA) followed by Bonferroni’s post hoc test. A significance level of *p* < 0.05 was set for all tests.

## 3 Results

### 3.1 Characterization of the scaffolds

#### 3.1.1 Physical and chemical properties of the scaffolds

The compressive strengths of the scaffolds prior to implantation differed significantly from each other (*p* < 0.001) [Mg225d: (6.00 ± 1.04) MPa, Mg225p: (14.12 ± 3.16) MPa, TCP: (1.95 ± 0.40) MPa].

**FIGURE 2:**
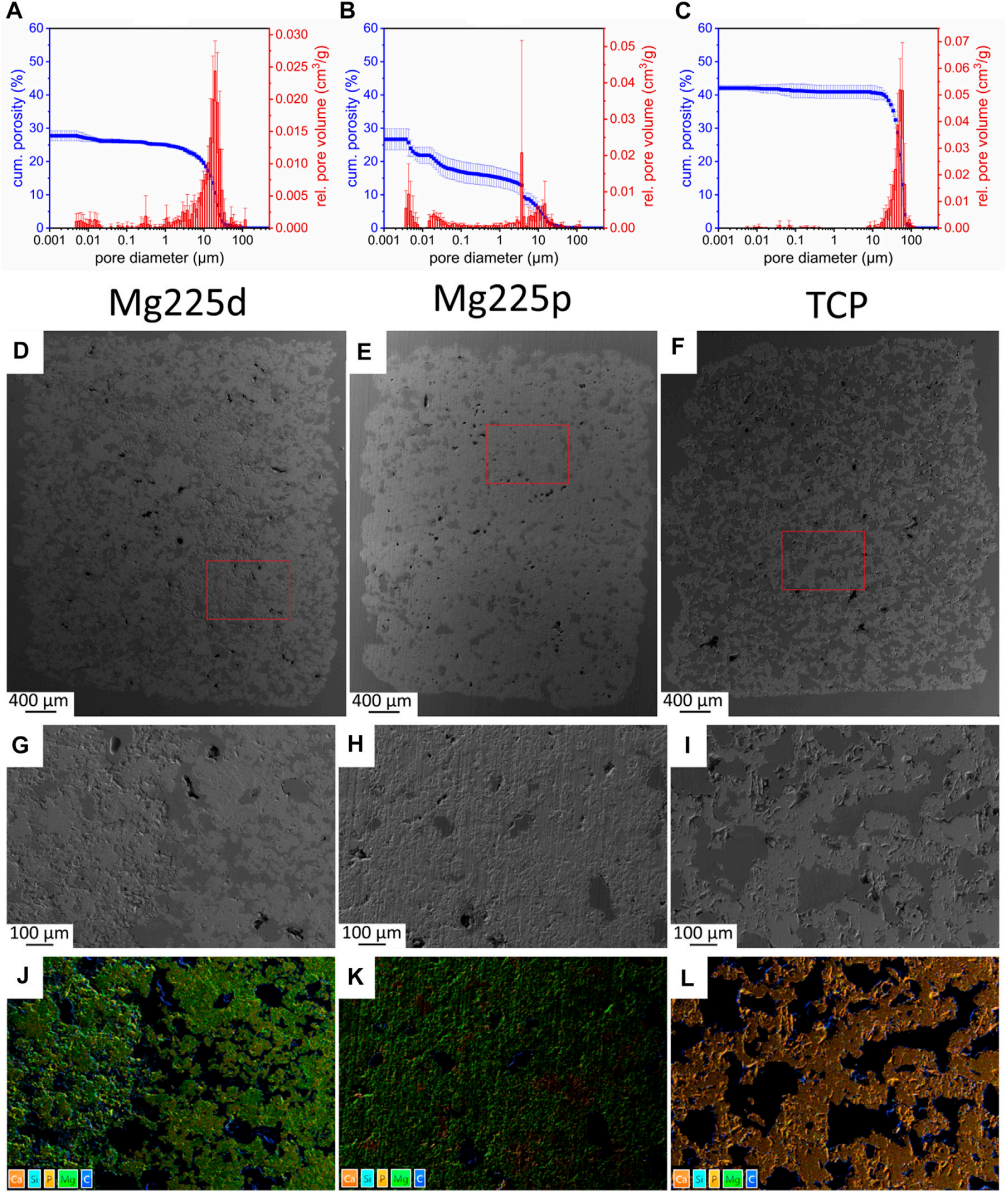
**(A–C)** Porosity, pore diameter and relative pore volume of **(A)** Mg225d, **(B)** Mg225p and **(C)** TCP. **(D–F)** SEM analysis (×24 magnification) of a **(D)** Mg225d, **(E)** Mg225p and **(F)** TCP scaffold prior to implantation with **(G–I)** ×100 magnification from the scaffold center of **(G)** Mg225d, **(H)** Mg225p and **(I)** TCP. **(D–I)** Dark gray areas: pores, light gray areas: scaffold material. **(J–L)** EDX analysis from the scaffold center of **(J)** Mg225d, **(K)** Mg225p and **(L)** TCP prior to implantation with the same position and magnification as **(G–I)**.

Mercury porosimeter analysis revealed an open porosity of (27.85 ± 1.67)% for Mg225d (Figure 2A). For Mg225p, an open porosity of (26.85 ± 3.02)% was determined (Figure 2B), while TCP had an open porosity of (42.11 ± 0.78)% (Figure 2C). The porosity of both CMPCs was significantly different compared to TCP (*p* < 0.001).

Due to the post-treatment of the scaffolds with DAHP (Mg225d) and PA (Mg225p), the following chemical reactions resulted in the partial transformation of stanfieldite and farringtonite into struvite (Mg225d (Eq. 1)), newberyite (Mg225p (Eqs. 2, 3)), and brushite (Mg225p (Eq. 3)), respectively.

$$2\;{Mg}_{3}{\left({PO}_{4}\right)}_{2}+3\;{\left({NH}_{4}\right)}_{2}{HPO}_{4}+36\;H_{2}0\;\to \;6\;{NH}_{4}{MgPO}_{4}\cdot 6\;H_{2}0+H_{3}{PO}_{4}\;\left(struvite\right)$$ (1)

$${Mg}_{3}{\left({PO}_{4}\right)}_{2}+H_{3}{PO}_{4}+9\;H_{2}0\;\to 3\;{MgHPO}_{4}\cdot 3\;H_{2}0\;\left(newberyite\right)$$ (2)

$${{Ca}_{4}Mg}_{5}{\left({PO}_{4}\right)}_{6}+3\;H_{3}{PO}_{4}+23\;H_{2}O\to 4\;{CaHPO}_{4}\cdot 2\;H_{2}0+5\;{MgHPO}_{4}\cdot 3\;H_{2}0\;\left(brushite,\;newberyite\right)$$ (3)

The quantitative chemical composition of the scaffolds in wt% is listed in Table 5.

**TABLE 5:** Chemical composition of Mg225d, Mg225p and TCP in wt%.

	Stan	Far	New	Bru	Stru	Periclas	α-TCP	β-TCP
Mg225d	59.84 ± 1.05	31.92 ± 1.05			5.91 ± 1.38	2.33 ± 0.53		
Mg225p	25.04 ± 1.29	13.19 ± 0.52	53.33 ± 0.69	8.42 ± 1.10				
TCP							1.49 ± 0.83	98.51 ± 0.83

Stan, Stanfieldit Ca_4_Mg_5_(PO_4_)_6_; Far, Farringtonit Mg_3_(PO_4_)_2_; New, Newberyit MgHPO_4_·3H_2_O; Bru, Brushit CaHPO_4_·2H_2_O; Stru, Struvit NH_4_MgPO_4_·6H_2_O; Periclas, MgO; α-TCP, alpha-Tricalciumphosphat Ca_3_(PO_4_)_2_; β-TCP, beta-Tricalciumphosphat Ca_3_(PO_4_)_2_.

#### 3.1.2 SEM and EDX analysis of the scaffolds before implantation

Analysis of the scaffolds by SEM and EDX showed an increasing porosity of the materials as listed: Mg225p < Mg225d < TCP. Mg225d showed large, interconnected pores as well as rough-appearing areas inside the scaffold, which probably contained unreacted raw powder (stanfieldite, farringtonite) (Figures 2D,G,J; Supplementary Figure S1A). EDX analysis also revealed a lower amount of Ca in the peripheral region of Mg225d than in the rough-appearing center, whereas Mg was homogeneously distributed. The matrix of Mg225p appeared more homogeneous and denser in EDX than in Mg225d (Figures 2E,H,K; Supplementary Figure S1B). The reddish areas visible in the EDX analysis (high Ca concentration) were probably comprised of brushite, which is a precipitation product of the reaction with the phosphoric acid (Figure 2K). The TCP scaffolds were composed of morphologically and chemically homogeneous CaP (Figures 2F,I,L; Supplementary Figure S1C).

### 3.2 Clinical examination

All rabbits were in good general condition following surgery for the duration of the respective observation periods. No animal showed signs of pain or lameness. Physiological wound healing occurred.

### 3.3 X-ray examination

**FIGURE 3:**
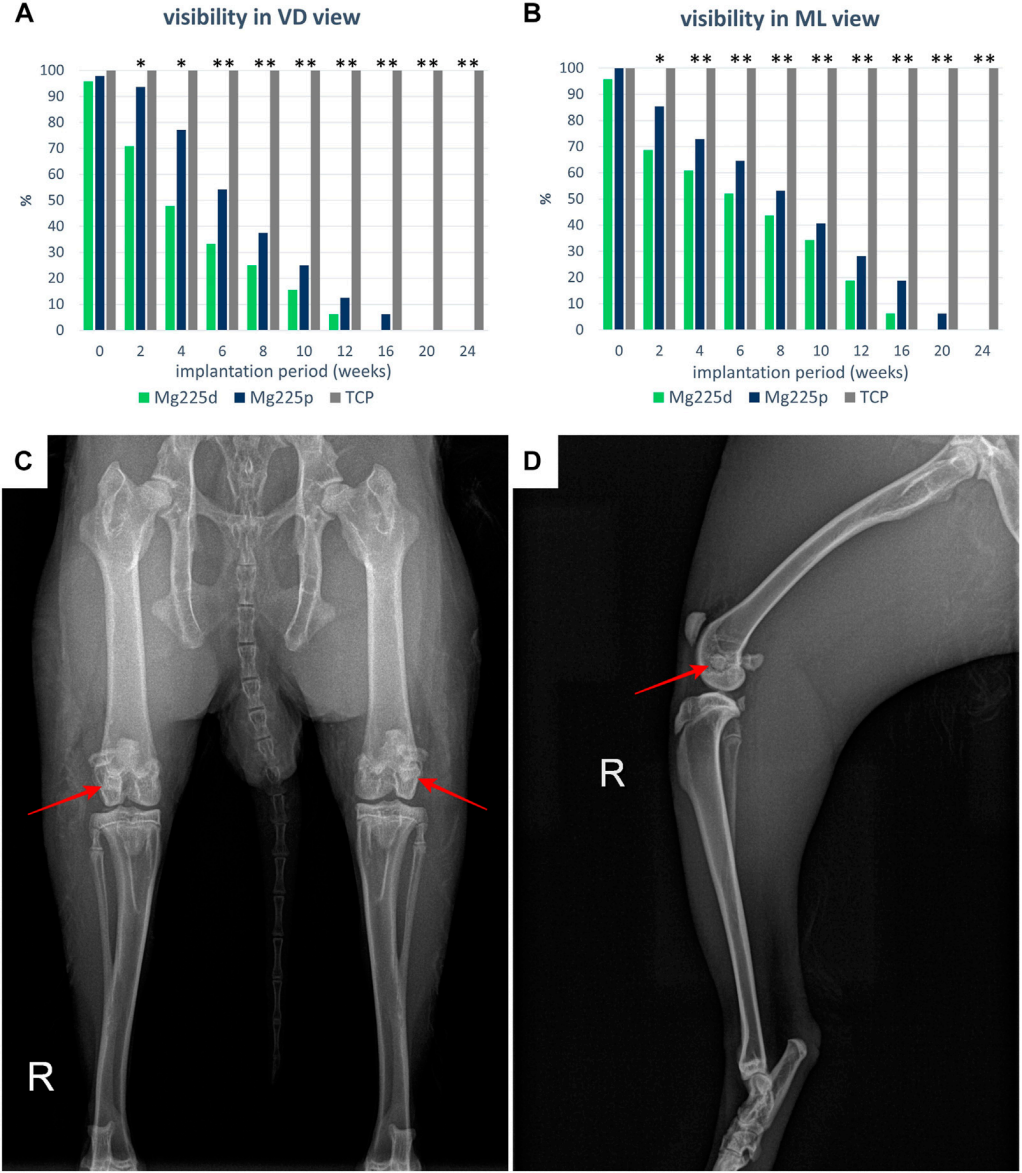
Visibility of the scaffolds in the X-ray in **(A)** ventrodorsal (VD) and **(B)** mediolateral (ML) view. Significant differences (*p* < 0.05) are marked with *: Mg225d—TCP, **: CMPCs—TCP. **(C)** VD X-ray view of the hind limbs directly after surgery with Mg225d implanted in the left and Mg225p in the right femoral condyle. **(D)** ML X-ray view of the right hind limb directly after surgery with implanted Mg225p scaffold.

Since Mg225d and Mg225p showed a radiopacity comparable to that of bone, some scaffolds could not be distinguished from the surrounding bone tissue already directly after surgery. Over the study period, the percentage of visible CMPC scaffolds rapidly decreased in both views and was significantly lower than with TCP from week 4 in ML view (*p* ≤ 0.042) and from week 6 in VD view (*p* < 0.001), respectively (Figure 3). The CMPC scaffolds were more difficult to delineate in the VD view due to overlap by the sesamoid bones, and therefore visible less frequent and for shorter periods (Mg225d up to week 12, Mg225p up to week 16) than in the ML view (Mg225d up to week 16, Mg225p up to week 20). All TCP scaffolds were clearly visible in both radiographic views at each examination time point.

### 3.4 *In-vivo* µCT examination

#### 3.4.1 Semi-quantitative evaluation of *in-vivo* µCT scans

**FIGURE 4:**
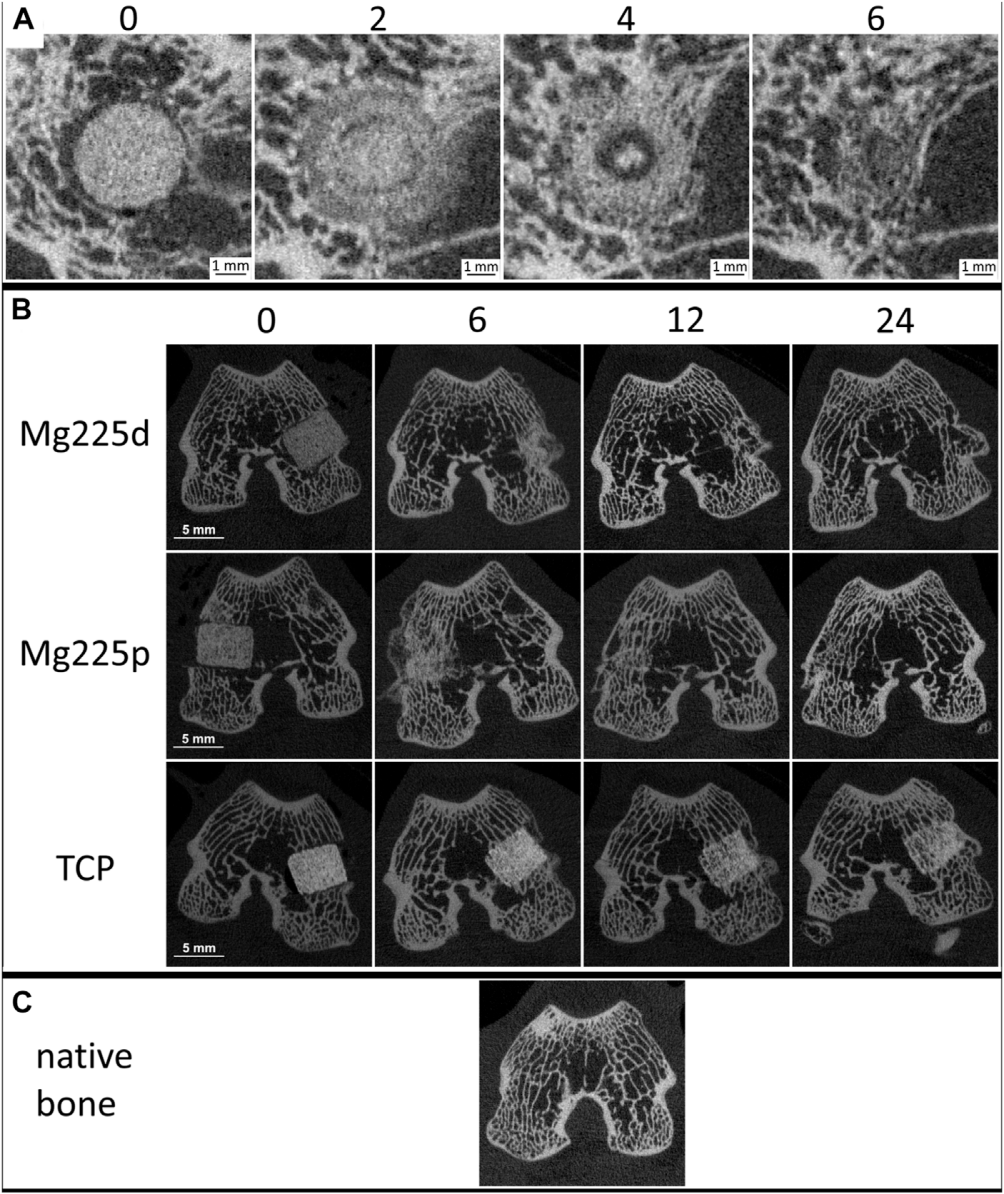
**(A)** *In-vivo* µCT images of Mg225d and surrounding cancellous bone in cross section over time (directly after surgery up to 6 weeks) with increasing scaffold-bone-contact and resorption zone. **(B)** *In-vivo* µCT images of the scaffolds (Mg225d, Mg225p and TCP) implanted in the distal femoral condyles over time (directly after surgery up to 24 weeks) **(C)** compared to native cancellous bone of the distal femoral condyle.

**FIGURE 5:**
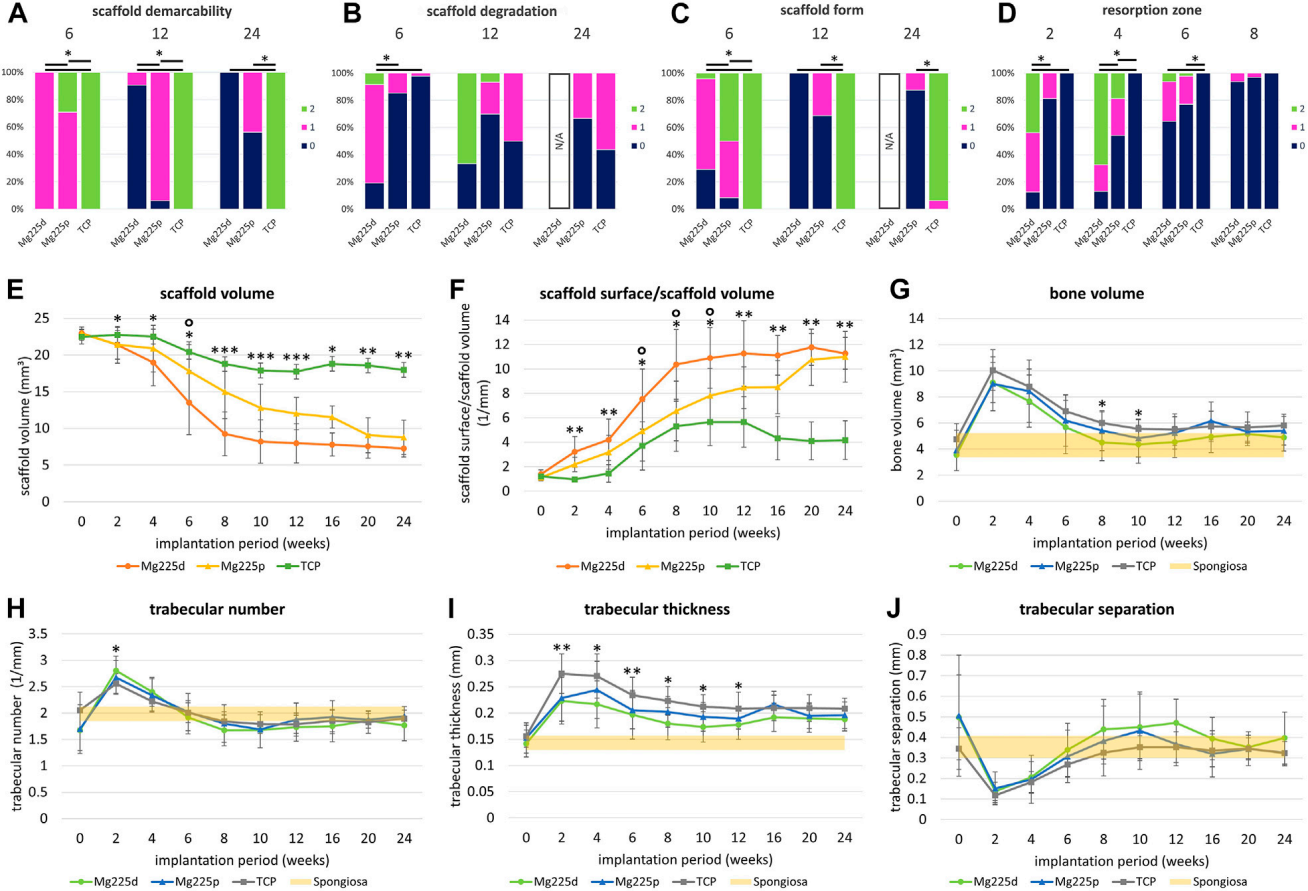
**(A–D)** Semi-quantitative *in-vivo* µCT evaluation of Mg225d, Mg225p and TCP. *: Significant differences (*p* < 0.05) between the individual materials. **(A)** Scaffold demarcability, **(B)** scaffold degradation and **(C)** scaffold form after 6, 12 and 24 weeks. Due to the lack of demarcability of all Mg225d scaffolds from the bone tissue from week 16 onwards, the evaluation of scaffold degradation and form was no longer possible at week 24. **(D)** Resorption zone after 2, 4, 6 and 8 weeks **(E–J)** Quantitative *in-vivo* µCT evaluation of the scaffolds [**(E)** scaffold volume (SV), **(F)** scaffold surface/scaffold volume (SS/SV)] and adjacent cancellous bone [**(G)** bone volume (BV), **(H)** trabecular number (Tb.N), **(I)** trabecular thickness (Tb.Th), **(J)** trabecular separation (Tb.Sp)] compared to native cancellous bone. Significant differences (*p* < 0.05) are marked with *: Mg225d—TCP, °: Mg225d—Mg225p, **: CMPCs—TCP, ***: all materials.

The demarcability of the CMPC scaffolds from surrounding bone decreased steadily over the 24-weeks study period (Figures 4, 5A; Supplementary Table S1). Between weeks 2 and 6 as well as at weeks 10 and 12, Mg225d was significantly less clearly demarcable from surrounding bone than Mg225p (*p* ≤ 0.023). From week 16 onwards, the demarcation of all Mg225d scaffolds was no longer possible. All TCP scaffolds were completely demarcable from the surrounding bone by week 24 and thus differed significantly from the CMPC scaffolds from week 6 onwards (*p* < 0.001).

The phenomenon of faster scaffold degradation on the side towards the bone marrow compared to the side towards the cortex was observed in all materials (Figure 5B; Supplementary Table S2). 6 and 12 weeks after surgery, at least half of the Mg225p and TCP scaffolds were equally degraded on the side towards the bone marrow and the cortex. At 24 weeks, more severe degradation was observed in the majority of TCP scaffolds on the side towards the bone marrow compared to the side towards the cortex. Mg225d exhibited significantly greater degradation close to the bone marrow than Mg225p and TCP between weeks 2 and 6 (*p* < 0.001). After 12 weeks, the majority of Mg225d scaffolds (67%) were no longer visible at the side towards the bone marrow.

Over the study period, the loss of cylindrical form of both CMPCs increased steadily and was significantly more pronounced than in TCP from week 4 onwards (*p* ≤ 0.003), which almost always exhibited a distinct cylindrical form until week 24 (Figures 4B, 5C; Supplementary Table S3). After 6 weeks, Mg225p (50% of scaffolds) still showed a distinct cylindrical form significantly more often than Mg225d (4% of scaffolds) (*p* < 0.001).

The CMPCs showed a resorption zone within the scaffolds between weeks 2 and 8, which was observed significantly more frequent and distinct in Mg225d than in Mg225p at weeks 2 and 4 (*p* < 0.001) (Figures 4A, 5D; Supplementary Table S4). In TCP, such a zone was indistinctly delineated in only a single scaffold at weeks 20 and 24.

Two weeks after surgery, several trabeculae were visible between the scaffold and the surrounding bone in the majority of scaffolds of all materials (Figure 4A, Supplementary Table S5). After 8 weeks at the latest, there was always a broad contact area existing between the scaffold and the surrounding bone.

#### 3.4.2 Quantitative evaluation of *in-vivo* µCT scans

Within the study period (immediately after surgery up to week 24), a significant decrease in SV was observed in the CMPCs (*p* < 0.001) (Figure 5E). The volume decrease was greatest for Mg225d (Mg225d: 68.47%, Mg225p: 61.75%, TCP: 20.14%). From week 2 onwards, the SV of Mg225d was significantly lower than that of TCP (*p* ≤ 0.042). Between weeks 6 and 12, Mg225d also had a significantly lower SV than Mg225p (*p* ≤ 0.049). From week 12 onwards, the CMPCs showed only a slight decrease in volume. In TCP, a significant decrease in SV was observed when comparing between week 12 and immediately after surgery (*p* = 0.002), which was followed briefly by a small increase in SV.

SS/SV increased significantly in the CMPCs over the study period of 24 weeks (*p* ≤ 0.001) (Figure 5F). In TCP, a significant increase was observed between immediately after surgery and week 12 (*p* = 0.009). For Mg225d, SS/SV was significantly higher than for TCP from week 2 onwards (*p* ≤ 0.001), for Mg225p this was the case at weeks 2 and 4 and from week 12 (*p* ≤ 0.049). Between weeks 6 and 10, the SS/SV of Mg225d was significantly greater than that of Mg225p (*p* ≤ 0.023).

BV in the scaffold environment increased slightly for all materials when comparing immediately after surgery with week 24 (Figure 5G). The significant increase in week 2 (*p* ≤ 0.005) was striking for all materials, followed by a continuous decrease until week 10 (Mg225d, Mg225p) and week 12 (TCP), respectively. At subsequent time points, the BV was within or slightly above the physiological range for cancellous bone at this site for all materials.

All materials showed an increase in the Tb.N at week 2, which was significant (*p* = 0.005) for the CMPCs (Figure 5H). This peak was followed by a continuous decrease until week 8 (Mg225d), week 10 (Mg225p) and week 12 (TCP), respectively. From week 6 onwards, the Tb.N was always within the physiological range for cancellous bone at this site or slightly lower.

After a significant increase until week 2 (Mg225d, TCP) or week 4 (Mg225p) (*p* ≤ 0.007), the Tb.Th decreased slightly in all materials (Figure 5I). From week 2 onwards, the Tb.Th was higher in every material than in cancellous bone at this site.

Tb.Sp was significantly lower in the CMPCs after 2 weeks than immediately after surgery (*p* ≤ 0.013) (Figure 5J). From week 2, the Tb.Sp increased again until week 10 (Mg225p) or week 12 (Mg225d, TCP) and was within or slightly above the physiological range for cancellous bone at this site from week 8 onwards.

### 3.5 µCT 80 examination

#### 3.5.1 Semi-quantitative evaluation of µCT 80 scans

**FIGURE 6:**
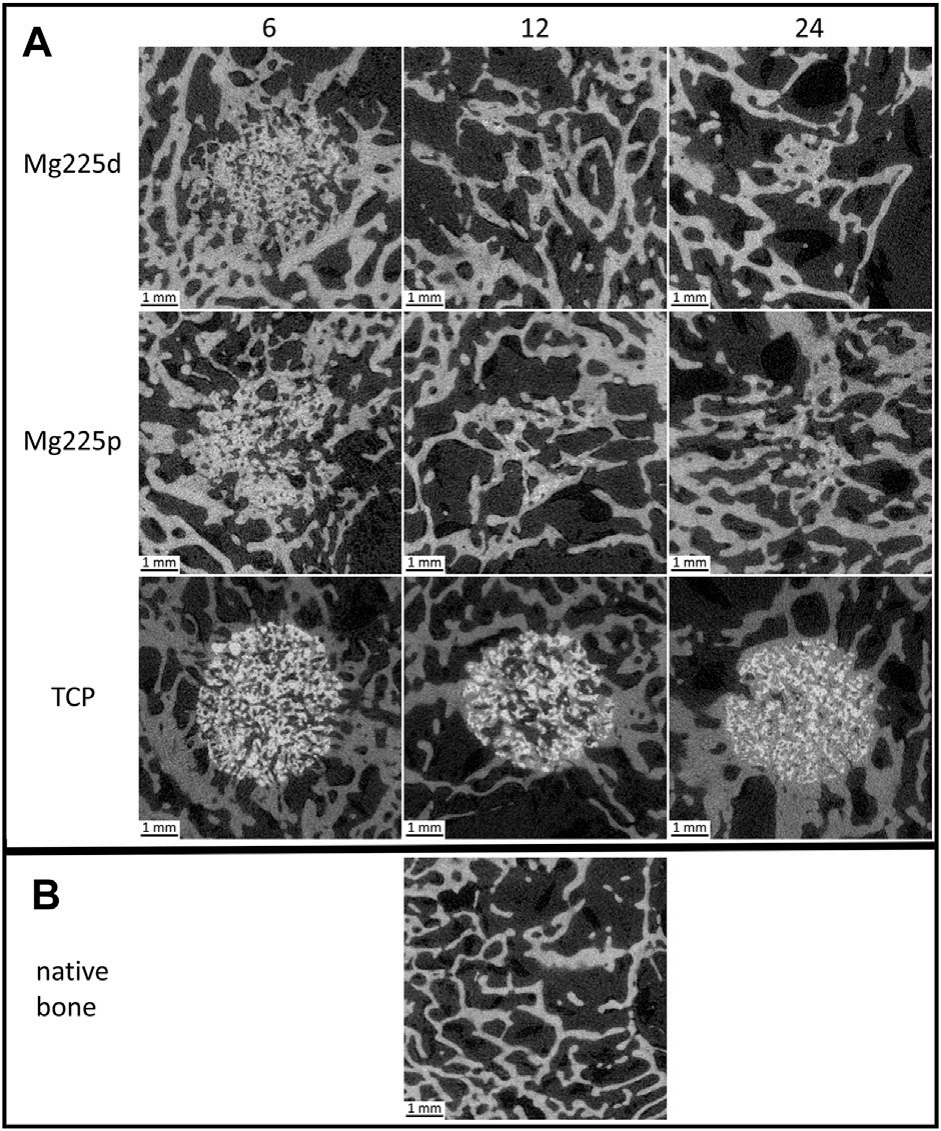
Cross-sectional µCT 80 images **(A)** of the scaffolds (Mg225d, Mg225p and TCP) implanted in the distal femoral condyles at 6, 12 and 24 weeks after surgery **(B)** compared to native cancellous bone of the lateral femoral condyle.

**FIGURE 7:**
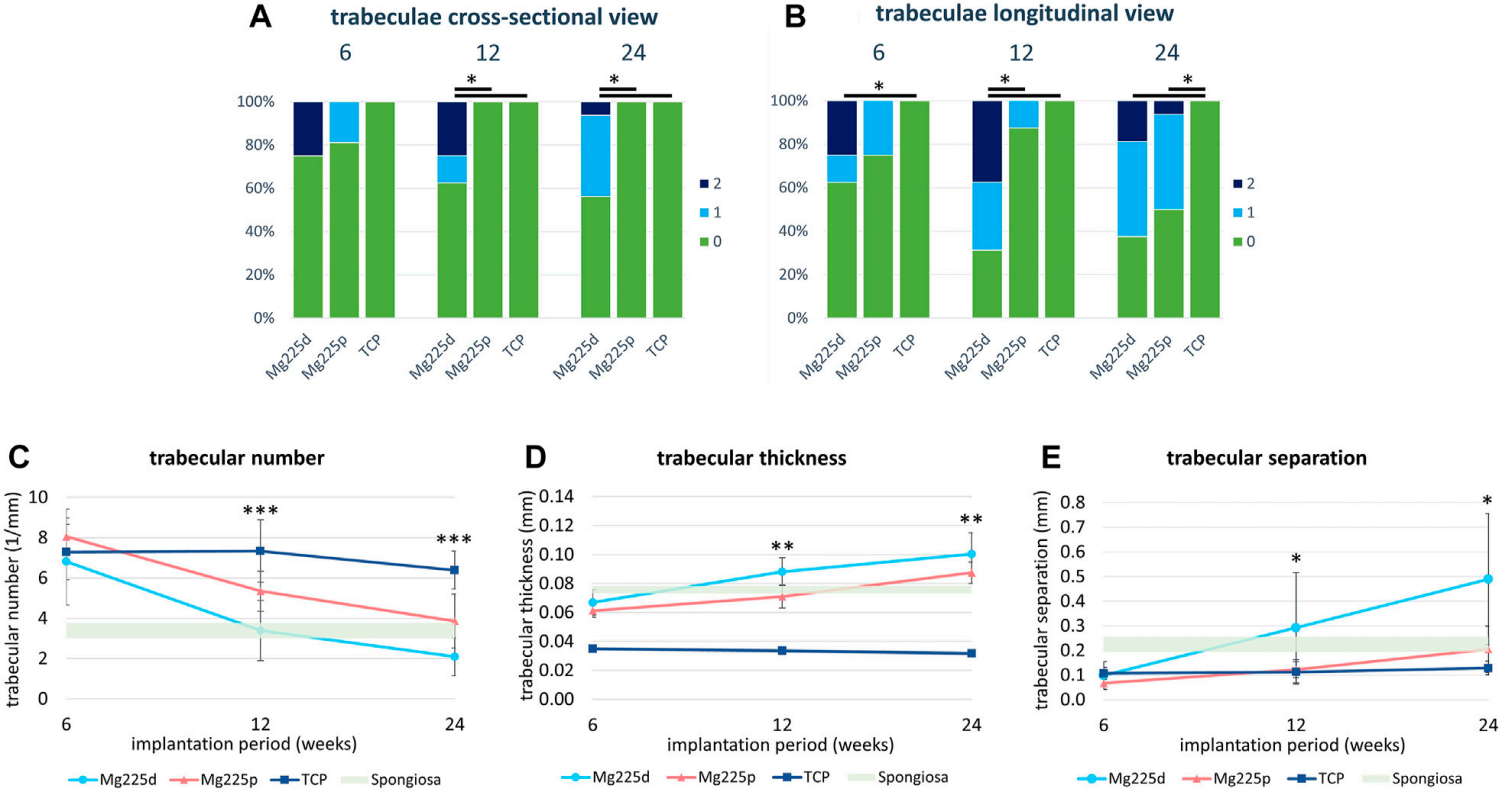
**(A,B)** Semi-quantitative µCT 80 evaluation of newly formed bone trabeculae within the initial scaffold volume **(A)** in cross-sectional view and **(B)** in longitudinal view 6, 12 and 24 weeks after surgery. *: Significant differences (*p* < 0.05) between the individual materials. **(C–E)** Quantitative µCT 80 evaluation of **(C)** trabecular number (Tb.N), **(D)** trabecular thickness (Tb.Th) and **(E)** trabecular separation (Tb.Sp) of newly formed bone trabeculae within the initial scaffold volume compared to native cancellous bone of the lateral femoral condyle. Significant differences (*p* < 0.05) are marked with *: Mg225d—TCP, **: CMPCs—TCP, ***: all materials.

After 6 weeks, all TCP and the majority of CMPC scaffolds showed trabeculae reaching into the center of the scaffold radius in cross section (Figures 6, 7A; Supplementary Figure S2). 12 and 24 weeks after surgery, new bone trabeculae had grown into the center of the scaffold radius in all Mg225p and all TCP scaffolds, whereas this was significantly less frequent in Mg225d (*p* ≤ 0.005).

In longitudinal section, at 6 weeks trabeculae were present and evenly distributed (same amount of trabeculae on the side towards the bone marrow as towards the cortex) throughout the scaffold volume in the majority of the CMPC scaffolds (Figure 7B). At 12 weeks, the proportion of Mg225p scaffolds with trabeculae evenly distributed throughout the scaffold volume increased, whereas Mg225d was significantly more likely to have a larger amount of trabeculae towards the cortex than towards the side of the bone marrow (*p* = 0.001). With TCP, the trabeculae were always evenly distributed throughout the scaffold volume, significantly differing this material from Mg225d at all observation time points (*p* ≤ 0.02).

#### 3.5.2 Quantitative evaluation of µCT 80 scans

Various changes of bone structure parameters were observed at the implantation sites over the study period. The Tb.N decreased significantly with the CMPCs between weeks 6 and 24 (*p* ≤ 0.013), while it decreased only slightly with TCP, resulting in significant differences of the Tb.N between all materials at weeks 12 and 24 (*p* ≤ 0.036) (Figure 7C).

The Tb.Th increased significantly in Mg225d and Mg225p between weeks 6 and 24 (*p* ≤ 0.001) and was always significantly higher with the CMPCs than with TCP (*p* ≤ 0.040) (Figure 7D).

A significant increase in Tb.Sp was observed in the CMPCs in contrast to TCP, when comparing week 6 with week 24 (*p* ≤ 0.002) (Figure 7E). At weeks 12 and 24, the Tb.Sp was significantly higher with Mg225d than with TCP (*p* ≤ 0.036).

### 3.6 Histological examination

#### 3.6.1 Semi-quantitative evaluation of histological sections

**FIGURE 8:**
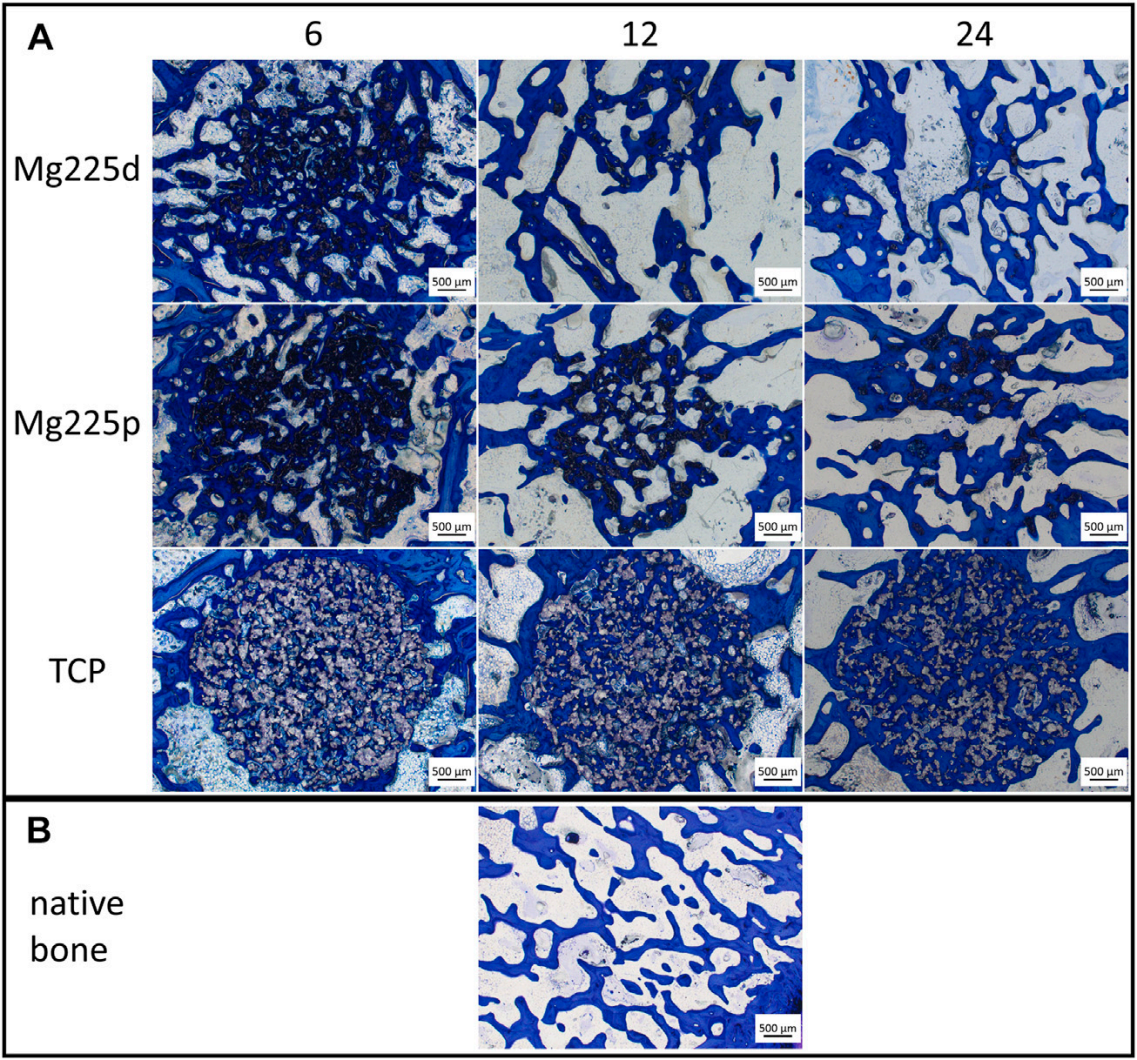
Histological thick sections (toluidine blue staining, magnification ×2.5/0.085) **(A)** from the center of the Mg225d, Mg225p and TCP scaffolds in cross section after 6, 12 and 24 weeks and **(B)** comparison to native cancellous bone of the lateral femoral condyle.

**FIGURE 9:**
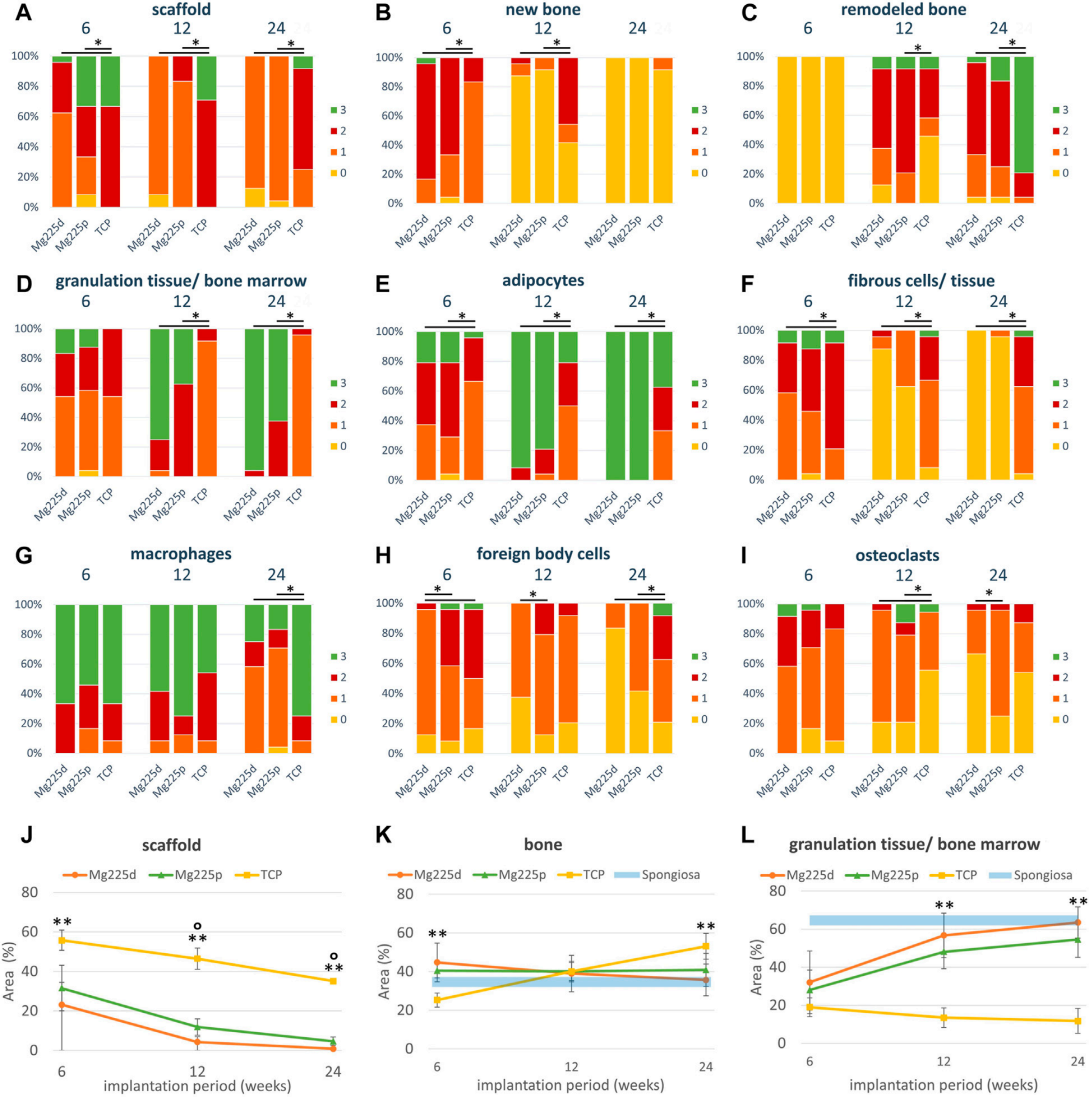
**(A–I)** Semi-quantitative histological evaluation of the scaffolds and the ingrowing tissue **(A–E)** at tissue level and **(F–I)** at cell level after 6, 12 and 24 weeks. *: Significant differences (*p* < 0.05) between the individual materials. **(J–L)** Histomorphometric measurement of the percentage area of **(J)** scaffold, **(K)** bone and **(L)** granulation tissue/bone marrow in the scaffold cross section (*Ø* = 1,060 pixels ≙ diameter of the OR of the semi-quantitative evaluation (*Ø* = 4.24 mm) ≙ scaffold diameter). Significant differences (*p* < 0.05) are marked with °: Mg225d—Mg225p, **: CMPCs—TCP.

Histologically, a centripetal directed degradation was visible in all materials, steadily increasing over the study period (Figures 8, 9A). At each observation time point, the percentage area of scaffold material in the scaffold cross section of the CMPCs differed significantly from TCP (*p* ≤ 0.041). The least material was always present from Mg225d, the most from TCP.

The material of all scaffolds was >50% surrounded by bone in the MR and OR after 6 weeks, in the IR this was observed in the majority (≥71.4%) of the scaffolds (Supplementary Figure S3A). At 6 weeks, significantly more bone had grown into the CMPC scaffolds than into TCP (*p* ≤ 0.004) (Figure 9B). At 12 and at 24 weeks, the proportion of new, immature bone steadily decreased, while the proportion of remodeled bone increased (Figures 9B,C). This remodeling of newly formed bone occurred more slowly in TCP than in the CMPCs, as evidenced by a still significantly greater amount of newly formed bone at week 12 in TCP compared to the CMPCs (*p* < 0.001). Nevertheless, after 24 weeks, TCP showed significantly more remodeled bone than the CMPCs (*p* < 0.001). With increasing bone maturity, the number of osteoblasts and the amount of osteoid at the newly formed bone trabeculae decreased over the course of the study in all materials (Supplementary Figures S3B,C). However, at week 24, TCP still exhibited significantly more osteoid than the CMPCs (*p* ≤ 0.017).

After 6 weeks, a low to moderate amount of granulation tissue/bone marrow (<50% of scaffold cross-sectional area) with a low to moderate number of adipocytes occurred in most scaffolds (Figures 9D,E). The percentage area of granulation tissue/bone marrow and the number of adipocytes increased markedly in the CMPCs over the study period. At each observation time point, the CMPCs showed significantly more adipocytes (*p* < 0.001) and at weeks 12 and 24 significantly more granulation tissue/bone marrow than TCP (*p* < 0.001). Mg225d always exhibited more granulation tissue/bone marrow than Mg225p. After 24 weeks, all CMPC scaffolds showed numerous adipocytes throughout the implantation area and in the majority of the CMPC scaffolds (Mg225d: 95.8%, Mg225p: 62.5% of ROIs), >50% of the scaffold cross-sectional area consisted of granulation tissue/bone marrow. In contrast, granulation tissue/bone marrow in TCP almost always accounted for a maximum of 25% of the scaffold cross-sectional area and in less than half of the ROIs many adipocytes were present after 24 weeks.

Except for the IR of one TCP scaffold, precursor cells were observed in all scaffolds of all materials in each ROI as early as after 6 weeks (Supplementary Figure S3D). An increase in their amount at 12 weeks was followed by a decrease in the amount of precursor cells at week 24 in all materials. Numerous blood vessels were present in all materials at each observation time point, with Mg225p always showing significantly greater vascularization than TCP (*p* ≤ 0.001) (Supplementary Figure S3E).

Connective tissue with fibrocytes was present in low to moderate amounts in the majority of scaffolds of all materials after 6 weeks (Figure 9F). In the CMPCs, the amount of connective tissue had decreased markedly at 12 weeks and after 24 weeks, it was no longer observed in Mg225d and only sporadically present in Mg225p. In TCP, however, significantly more connective tissue with fibrocytes than in the CMPCs was observed at each observation time point (*p* < 0.001). In all materials, connective tissue was almost always more abundant in the IR than in the MR and OR. A resorption zone (annular zone within the scaffold cross section, containing many cells and connective tissue) was observed after 6 weeks in 25% of the Mg225d scaffolds and in 12.5% of the TCP scaffolds in the IR (score 1) and after 24 weeks in 12.5% of the TCP scaffolds in the MR (score 1) and the IR (score 2) (scoring system in Table 4), respectively (Supplementary Figure S3F).

In the CMPCs, many macrophages were observed in >54% of ROIs at weeks 6 and 12 (Figure 9G). After 24 weeks, only a small or moderate number of macrophages was present in ≥75% of ROIs in the CMPCs. As many macrophages were observed in TCP throughout the study period, TCP differed significantly from the CMPCs at week 24 (*p* ≤ 0.001).

At weeks 6 and 12, few FBCs were present in ≥50% of the ROIs of the CMPC scaffolds (Figure 9H). In Mg225p, they were significantly more frequent than in Mg225d (*p* ≤ 0.017). In TCP, moderate to many FBCs were present at each observation time point (week 6: 50%, week 12: 8.3%, week 24: 37.5% of ROIs), differing it significantly from the CMPCs at week 24 (*p* ≤ 0.042). Overall, however, the number of FBCs decreased over the study period for all materials.

After 6 weeks, moderate to many osteoclasts were present in 41.7% of ROIs in Mg225d and 29.2% of ROIs in Mg225p, and their amount decreased with increasing implantation time (Figure 9I). TCP showed fewer osteoclasts than the CMPCs at 6 weeks and significantly fewer at 12 weeks (*p* ≤ 0.018). After 24 weeks, no or few osteoclasts were observed in ≥87.5% of ROIs in all materials.

#### 3.6.2 Quantitative evaluation (histomorphometry)

All scaffolds showed a significant material loss over the study period (*p* ≤ 0.003) (Figure 9J). After 24 weeks, the CMPC scaffolds were almost completely degraded (percentage of area of scaffold material in the scaffold cross section: Mg225d: 0.85%, Mg225p: 4.63%), while the remaining TCP material comprised 35.14% of the scaffold cross-sectional area. There was always significantly less material present from Mg225d and Mg225p than from TCP (*p* < 0.001). Additionally, in weeks 12 and 24, there was significantly less material present from Mg225d than from Mg225p (*p* ≤ 0.007).

After 6 weeks, significantly more bone had grown into the CMPC scaffolds than into the TCP scaffolds (*p* ≤ 0.001) (Figure 9K). After 12 weeks, the percentage of bone was approximately the same for all materials. After 24 weeks, it was significantly higher in TCP than in the CMPCs (*p* ≤ 0.022).

Over the study period, the amount of soft tissue (granulation tissue, bone marrow) increased significantly in Mg225d and Mg225p, resulting in significantly more soft tissue with the CMPCs than with TCP at weeks 12 and 24 (*p* < 0.001) (Figure 9L).

### 3.7 SEM and EDX analysis of the unstained histological sections

**FIGURE 10:**
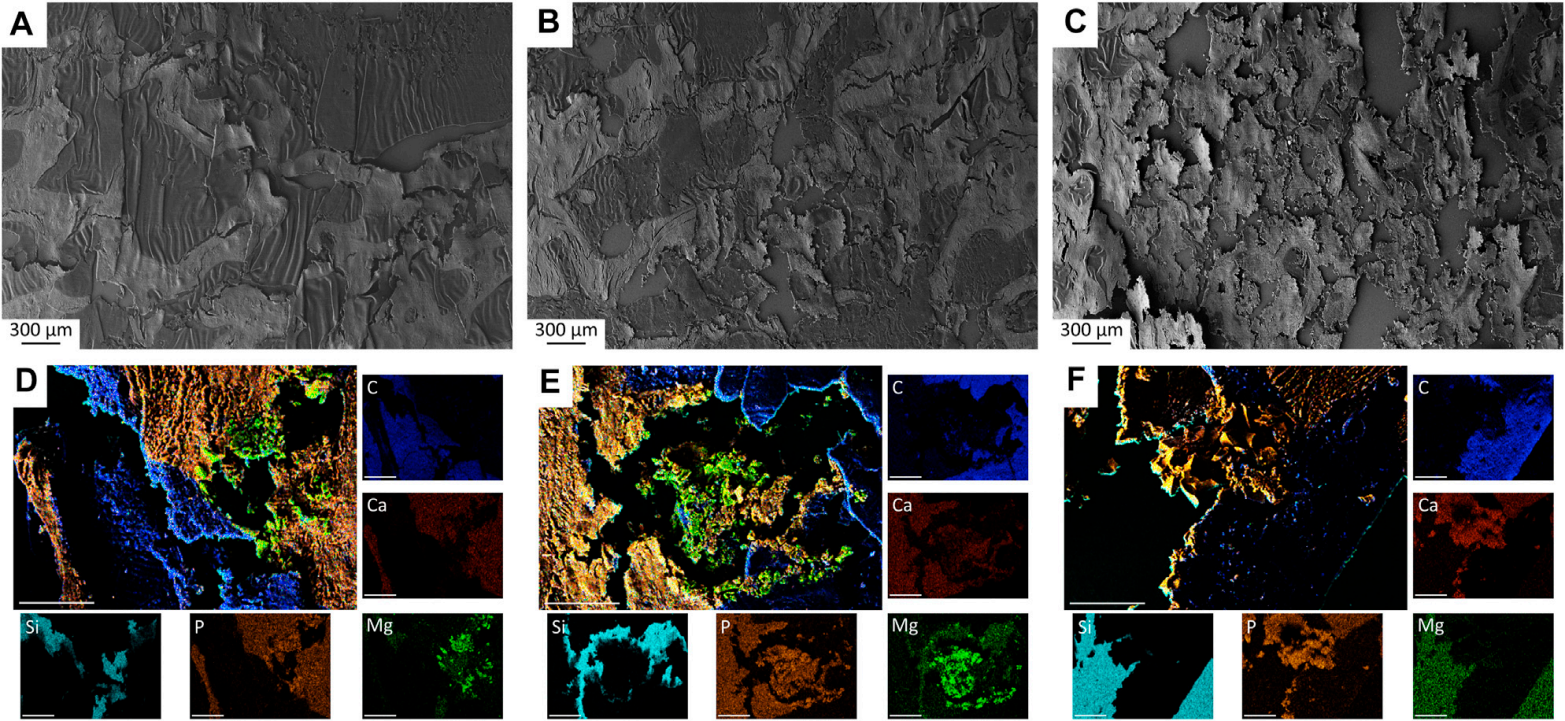
**(A–C)** SEM analysis of histological thin sections (×28 magnification) with **(A)** Mg225d, **(B)** Mg225p and **(C)** TCP scaffold incorporated by newly formed cancellous bone after 12 weeks. **(D–F)** EDX analysis of histological thin sections (×500 magnification) with **(D)** Mg225d, **(E)** Mg225p and **(F)** TCP particles incorporated by newly formed cancellous bone after 12 weeks. Scale bar = 50 µm.

Small scaffold particles could be detected in SEM and EDX analysis in all materials at all observation time points (Figure 10; Supplementary Figures S4, S5). A continuously increasing material degradation was observed. The material residues appeared as small, sharp-edged particles that were excellently integrated into the surrounding bone. After only 6 weeks, the entire implantation area of Mg225d was infiltrated by bone (Supplementary Figure S4A). The scaffold structure was no longer recognizable, the material was largely replaced by newly formed bone and the remaining particles were enclosed by it (Supplementary Figure S4D). After 12 weeks, the scaffolds were already degraded to a large extend, and after 24 weeks, only smallest particles of Mg225d were still present, incorporated into bone trabeculae (Figure 10A,D; Supplementary Figures S5A,D). The degradation of Mg225p showed a similar course, with always slightly more material present than with Mg225d. From week 12 onwards, the scaffold structure was no longer evident in Mg225p either. As with Mg225d, the scaffold was completely traversed by new bone trabeculae after 6 weeks. Mg225p also showed an excellent osseointegration and almost complete degradation after 24 weeks (Figure 10B,E; Supplementary Figures S4B,E, S5B,E). A greater amount of cement matrix was present with TCP than with the CMPCs at each time point examined, and less extensive material degradation was observed over time. As of 6 weeks, the entire implant site was also infiltrated by new bone. As with the CMPCs, good osseointegration of the scaffold material was observed throughout the study period (Figure 10C,F; Supplementary Figures S4C,F, S5C,F).

## 4 Discussion

Due to their better mechanical properties as well as their faster degradation compared to CPCs, CMPCs have been increasingly researched in recent years in the form of cement pastes and granules (Wu et al., 2008a; Wu et al., 2008b; Klammert et al., 2010a; Jia et al., 2010; Wei et al., 2010; Vorndran et al., 2011; Zeng et al., 2012; Ewald et al., 2019; Fuchs et al., 2021; Götz et al., 2021). However, for the treatment of many bone defects, three-dimensional, dimensionally stable scaffolds are required, that can be produced patient-specifically. Therefore, 3D powder printing has emerged as a promising manufacturing technique for bone substitutes (Castilho et al., 2014a; Roseti et al., 2017; Zhang et al., 2019). This study varied the post-treatment of 3D powder printed CMPC scaffolds produced from the ceramic cement powder Ca_0.75_Mg_2.25_(PO_4_)_2_ by either immersion in DAHP (alkaline post-treatment) or by infiltration with PA (acid post-treatment), affecting the physical and chemical properties of the scaffolds. The influence of DAHP (Mg225d) or PA (Mg225p) post-treatment on biocompatibility, osseointegration and degradation behavior of the scaffolds was investigated *in vivo* and compared between the two materials. Scaffolds of the established material TCP served as reference. The scaffolds were implanted into the lateral femoral condyles of rabbits and assessed by regular clinical and radiological (X-Ray and µCT) examinations up to 24 weeks. After euthanasia of the animals, higher-resolution µCT 80 and histological examinations were performed on the explanted scaffold-bone-complexes, as well as an analysis by SEM and EDX.

When the compressive strength of the scaffolds was investigated prior to implantation, all materials differed significantly from each other, with TCP having the lowest compressive strength and Mg225p the highest. The examination of the porosity with the mercury porosimeter as well as SEM analysis of the scaffolds before implantation showed that TCP had the highest porosity and Mg225p the lowest. As an increase in pore size and porosity affects the structural integrity of the scaffolds and reduces their mechanical properties (Karageorgiou and Kaplan, 2005), an inverse relationship between porosity and compressive strength has been described in the literature (Vorndran et al., 2011; Zhang et al., 2014; Wang et al., 2019), which was also observed with the materials investigated in this study.

As previously described in studies on CMPCs with the application form of granules, pastes or cylindrical scaffolds (Wu et al., 2008b; Wei et al., 2010; Ewald et al., 2019; Fuchs et al., 2021; Götz et al., 2021; Kowalewicz et al., 2021), the CMPCs investigated in this study as well as TCP showed excellent clinical tolerability with physiological wound healing. No animal showed lameness or pain.

Radiographically, the visibility of the CMPC scaffolds decreased rapidly and was significantly lower than that of TCP in both views from week 6 at the latest, whereas the latter was always still clearly delineable from surrounding bone until week 24. The longer visibility of the TCP scaffolds can be explained by the higher content of radiopaque Ca. However, it could also be an indication of a slower degradation compared to the CMPC scaffolds, which Fuchs et al. (2021) also observed when investigating granules of CMPCs and CPC (HA) in a rabbit model.

The CMPCs showed a significant volume loss as well as a significant increase of the surface area to volume ratio upon *in-vivo* µCT examination over the study period. The volume loss was significantly more pronounced with the CMPCs than with TCP. The significantly faster volume degradation of Mg225d compared to Mg225p can be explained by the different chemical and physical properties of the scaffolds. The chemical solubility of the individual phases of the CMPCs increases as listed: farringtonite < struvite < brushite < newberyite (Ostrowski et al., 2016). No data on the solubility of stanfieldite are available in the accessible literature. The cement raw materials farringtonite and stanfieldite are mainly cohered by the binder phases struvite (Mg225d), newberyite and brushite (Mg225p) formed during post-treatment with DAHP and PA, respectively (cf. Eqs. 1–3 in Section 3.1.1). The importance of the wt% fraction of the binder phase on the chemical degradation rate has previously been shown in the *in-vitro* study on scaffolds of farringtonite (76 wt%) and struvite (24 wt%) (TMP-D) and farringtonite (57 wt%) and newberyite (43 wt%) (TMP-P), respectively (Gefel et al., 2022). Despite the higher proportion of the chemically less soluble phase farringtonite, a faster and greater chemical solubility of TMP-D was observed compared to TMP-P. This phenomenon was based on the faster degradation of the low proportion of the binder phase struvite (24 wt%) compared to newberyite (43 wt%), as a result of which the degradation of the cement raw materials proceeded more rapidly (Gefel et al., 2022). In the present study, the same process can be assumed. In Mg225d, the binder phase struvite was probably degraded relatively quickly due to its low wt% content (<6 wt%). Based on the SEM and EDX analysis of Mg225d, it can also be assumed that due to the lower Ca-occurrence, more struvite and less stanfieldite was present in the peripheral region of the scaffolds, while areas of unreacted raw powder with low mechanical strength were probably present in the scaffold center. As soon as the outer areas with more struvite had dissolved, the scaffolds lost stability, the remaining cement matrix was no longer sufficiently cohered and rapidly disintegrated into individual particles, which was particularly accelerated by the ingrowth of bone and cells. Despite the higher chemical solubility of newberyite and brushite, the degradation of these binder phases took more time in Mg225p due to the higher wt% content (63.75 wt%) as well as the uniform distribution. Consequently, the scaffold matrix was cohered for a longer time and the degradation was slower than for Mg225d. Since dissolution is a physico-chemical process, it is controlled not only by solubility but also by porosity, pore size and surface area to volume ratio (Dorozhkin, 2013). SEM and EDX analysis of the scaffolds prior to implantation showed that Mg225d had large (Ø > 100 µm), interconnected pores, which were not detected in the mercury porosimeter measurement because they were either occluded or beyond the accessible measurement range. Therefore, the porosity and pore size of Mg225d can be assumed to be much larger than in Mg225p. Wei et al. (2010) observed significantly greater weight degradation for CPC and MPC scaffolds with higher microporosity and larger surface area in simulated body fluid (SBF) solution. Also, Kim et al. (2016) observed a faster material degradation with larger pore size during *in-vivo* investigation of MPC scaffolds. Therefore, it can be assumed that the higher porosity and larger pore size of Mg225d compared to Mg225p investigated in this study had an additional accelerating effect on the degradation rate. The solubility of the scaffolds was also likely enhanced by an increase in the scaffold surface area to volume ratio (SS/SV), which was accompanied by the loss of the cohesive scaffold structure. The significantly larger SS/SV between weeks 6 and 10 for Mg225d compared to Mg225p confirms that the Mg225d scaffolds disintegrated into individual material particles much faster than Mg225p, which further accelerated the degradation of Mg225d. Since TCP has a relatively low solubility (Wang and Nancollas, 2008; Dorozhkin, 2013), dissolution of the TCP scaffolds investigated in this study occurred only to a very small extent.

Due to the very comparable radiopacity of the scaffolds and bone, distinct differentiation between scaffold material and bone that had grown into the implantation area was not always possible in *in-vivo* µCT. In this regard, difficulties have also occurred in various other *in-vivo* studies on CaPs, CPCs or MPCs (Chopra et al., 2009; Huber et al., 2009; Kasuya et al., 2012; Kanter, 2014). Therefore, it is likely that newly formed bone was erroneously attributed to the scaffold volume, especially at the later observation time points, which also explains the increase in SV at TCP once again between weeks 12 and 16. However, the histomorphometric examinations in the present study clearly demonstrated the almost complete degradation of the CMPC scaffolds after 24 weeks.

The faster scaffold degradation on the side towards the bone marrow observed in the CMPCs in the present study has also been described by other authors for magnesium-based implants. It is assumed that degradation progresses more rapidly on the side towards the medullary cavity due to the higher blood vessel supply and the weaker trabecular network in the scaffold environment (Xu et al., 2007; Höh et al., 2009; Zhang et al., 2009). In the present study, it can be assumed that due to the overall faster degradation of Mg225d compared to Mg225p, increased material degradation on the side towards the medullary cavity was significantly more pronounced in Mg225d than in Mg225p.

Both CMPCs as well as TCP investigated in this study showed rapid and comprehensive osseointegration in the *in-vivo* µCT examination by the presence of a broad direct scaffold-bone-contact after 8 weeks at the latest. This indicates optimal surface properties as well as excellent biocompatibility and osteogenesis of the scaffolds. The formation of an extensive direct bone-implant contact has also been demonstrated in other studies on CMPCs (Wu et al., 2008b; Wei et al., 2010; Ewald et al., 2019; Fuchs et al., 2021). The high microporosity of the 3D powder-printed scaffolds compared to cement pastes results in a large surface area, which has a positive effect on the integration into the surrounding bone. Various *in-vivo* studies have shown that improved osteogenesis and osseointegration occur with porous compared to solid implants, as pores allow cell migration and proliferation as well as vascularization (Karageorgiou and Kaplan, 2005). *In-vitro* investigations of CPC and MPC scaffolds have shown that the presence of micropores in particular has a positive effect on cell growth (Wei et al., 2010). A porous surface also improves mechanical interlocking and stability at the critical interface between scaffold and surrounding bone (Karageorgiou and Kaplan, 2005).

In the present study, the significant increase in BV observed in the adjacent scaffold environment at week 2 for all materials was striking, suggesting an osteoconductive and even possible osteoinductive effect of the materials. Kanter (2014) also observed an increased BV in the implant environment compared to peripheral bone when studying CPCs and MPCs in a sheep model. However, she also observed increased bone formation in direct proximity around empty borehole defects. Albrektsson and Johansson (2001) assumed that even a trauma to the bone results in osteoinduction. Therefore, it is also possible that in the present study, increased BV and osteoinduction in the scaffold vicinity was only induced by the trauma of drilling. However, as no comparative empty borehole defect was assessed in the present study, this phenomenon could not be clarified conclusively. The maximum increase in BV at week 2 was followed by a continuous decrease in BV until week 10 (CMPCs) or week 12 (TCP) and an approach to the physiological volume of cancellous bone. An osteoconductive and even slightly osteoinductive effect of the materials investigated in this study is nevertheless suspected, as the BV in the immediate vicinity of the CMPCs as well as of TCP was always slightly higher or in the upper reference range of physiological bone at this location. It is known, that the release of ions from degrading implants can greatly influence the formation of new bone around an implant (Ostrowski et al., 2016). A high Mg-concentration promotes osteoblast proliferation and differentiation and increases their activity (Wu et al., 2015; He et al., 2016) and a local increase of Ca- and PO_4_ -ions to a supra-physiological level has a positive effect on new bone formation (Chai et al., 2012). The higher BV in the vicinity of TCP at 24 weeks is probably caused by the higher Ca-content of the material compared with the CMPCs and the higher amount of remaining scaffold material releasing ions until the end of the study*.* The osteoconductive character as well as the promotion of osteogenesis and bone regeneration by CMPCs has yet been described by other authors (Wu et al., 2008b; Wei et al., 2010; Ewald et al., 2019; Fuchs et al., 2021). Studies on MPCs (Kim et al., 2016; Ostrowski et al., 2016; Kanter et al., 2018; Nabiyouni et al., 2018; Sarkar et al., 2019) and TCP (Walsh et al., 2008; Samavedi et al., 2013; Bohner et al., 2020) also demonstrated an osteoconductive behavior of these materials as well as the stimulation of bone formation.

In the present study, a decrease in Tb.N as well as an increase in Tb.Th and Tb.Sp over the study period was observed for all materials in the *in-vivo* µCT in the scaffold vicinity, as well as for the CMPCs in the higher-resolution µCT 80 within the initial scaffold volume. This progression was also observed by Kanter et al. (2018) when investigating MPCs. It suggests that the newly formed bone remodeled, adapted to the physiological situation and matured over the study period, resulting in the renewed presence of nearly physiological cancellous bone within the initial scaffold volume of Mg225p after 24 weeks. By week 24, a high Tb.N with small Tb.Th and Tb.Sp occurred within the scaffold volume of TCP, revealing a slower speed of bone remodeling, possibly because the large amount of remaining material spatially inhibited trabecular growth and bone maturation. Additionally, µCT 80 examinations of the scaffold longitudinal sections showed that the newly formed bone trabeculae within the initial scaffold volume were mostly located in the implantation area close to the cortex in Mg225d, whereas they were more evenly distributed in Mg225p with regard to their localization. This can be explained by the fact that Mg225d degraded significantly faster on the side towards the bone marrow than Mg225p and that new bone formation close to the bone marrow could not follow the degradation rate of the scaffold. Ewald et al. (2019) observed a decrease in bone-implant-contact in the faster degrading material when comparing 6 to 12 weeks during the *in-vivo* investigation of CMPC pastes and also assumed too rapid material degradation as a reason. In Mg225p, the material particles probably served as a guide for the new bone for a longer period of time, allowing a more uniform trabecular network to be formed.

In histology, all scaffolds examined in the present study quickly showed good osseointegration and replacement by newly formed trabecular bone increasing over time. Within the observation period, the amount of immature bone and osteoid and the number of osteoblasts in the initial implantation area of all materials decreased, while simultaneously the amount of remodeled, mature bone increased, which is typical for bone maturation (Hadjidakis and Androulakis, 2006; Katsimbri, 2017). The large amount of precursor cells in the bone marrow observed mainly after 12 weeks was also studied by Kanter et al. (2018). It can be considered as an active state of cell organization, resulting in centripetally directed formation of trabecular bone (Kanter et al., 2018). In TCP, significantly more immature bone after 12 weeks and significantly more osteoid after 24 weeks than in the CMPCs was still observed. This suggests that bone maturation occurred more slowly with TCP and was not yet completed at the end of the study period. The significantly higher proportion of bone in TCP compared to the CMPCs after 24 weeks is in contradiction with results from other *in-vivo* studies on CPCs and CMPCs in rabbits (Wu et al., 2008b; Wei et al., 2010). As bone ingrowth is facilitated by increased porosity and pore size (Karageorgiou and Kaplan, 2005), the greater amount of bone observed with TCP in the present study could be attributed to the significantly higher porosity of TCP compared to the CMPCs.

Within the implantation area of the CMPCs, the amount of granulation tissue rich of blood vessels and cells as well as the number of adipocytes increased continuously over the study period. At the same time, the amount of connective tissue with fibrocytes decreased markedly, resulting in no (Mg225d) or only sporadic (Mg225p) appearance after 24 weeks. This indicates that the ingrowing tissue has transformed into mature, physiological bone marrow, as this is also described in the literature (Travlos, 2006; Horowitz et al., 2017; Nombela-Arrieta and Manz, 2017; Lucas, 2021). TCP demonstrated significantly less granulation tissue/bone marrow at weeks 12 and 24 and at each observation time point significantly more connective tissue with fibrocytes than the CMPCs. It is likely that in TCP, due to the slower bone maturation, only little bone marrow was yet formed within the numerous small fibrovascular islets over the study period of 24 weeks. Possibly, the high amount of bone observed with TCP slowed down this remodeling process due to the spatial restriction of the granulation tissue. However, since complete bone replacement with restitutio ad integrum of the bone tissue is described in the literature for TCP (Wiltfang et al., 2002; Horch et al., 2006; Kolk et al., 2012), the formation of physiological bone marrow is also likely with a longer observation time.

FBCs express important growth factors for new blood vessel formation and a correlation between the amount of FBCs and the vascularization rate was observed in a study on TCP granules (Ghanaati et al., 2010; Al-Maawi et al., 2021), which could not be confirmed in the present study. Even though moderate to many FBCs were frequently present with TCP, at each observation time point significantly fewer blood vessels occurred within the implantation area of TCP than with Mg225p. Possibly, this could also be due to the spatial limitation of bone and scaffold material with TCP.

For all materials in the present study, centripetal directed degradation was observed in the histological examination, continuously increasing over the course of the investigation. The CMPCs differed significantly from TCP at each observation time point and were almost completely degraded after 24 weeks, whereas numerous particles of TCP were still present at the end of the study. As supposed for the *in-vivo* degradation of CMPCs by Wu et al. (2008b) and Wei et al. (2010), a two-step degradation mechanism is also assumed for the CMPC scaffolds investigated in this study. The previously described first step of chemical dissolution of the cements during the early implantation time resulted in surface enlargement and thus alteration of the microstructure of the scaffolds, which likely facilitated the cell-mediated resorption that occurred later in the second step. *In-vivo* degradation of CaP-based biomaterials is also thought to occur by a combination of chemical dissolution and cell-mediated resorption (Theiss et al., 2005; Dorozhkin, 2013). For rapidly resorbable CPCs such as brushite, mainly macrophages and FBCs are involved in the resorption process, whereas slowly resorbable CPCs such as apatite are degraded by osteoclasts (Apelt et al., 2004; Theiss et al., 2005; Dorozhkin, 2008, 2013). For MPCs, passive resorption by chemical dissolution in magnesium and phosphate ions has been described (Klammert et al., 2011; Kim et al., 2016). Gefel et al. (2022) could not detect an involvement of osteoclasts in the degradation of MPCs in *in-vitro* studies. In various *in-vivo* studies, however, osteoclasts were observed at the implantation site of MPCs, indicating a possible active cellular resorption as well (Zeng et al., 2012; Kim et al., 2016; Kanter et al., 2018). In the present study, both numerous macrophages and multinucleated cells located directly at the scaffold material were observed. The number of macrophages decreased strongly in the CMPCs with advanced implantation time and degradation of the scaffolds, suggesting that they were involved to a large extent in material degradation. The FBCs observed with the CMPCs especially at the early observation time points were probably also involved in the cellular degradation. The significantly more frequent occurrence of FBCs at weeks 6 and 12 in Mg225p than in Mg225d correlates with the still greater amount of remaining scaffold material in Mg225p at these time points and supports this hypothesis. Furthermore, since with the CMPCs, slightly (week 6) or even significant (week 12) more osteoclasts were observed than with TCP, it is reasonable to assume that they were also participating in the degradation of the MPC phases. However, the increased occurrence of osteoclasts with the CMPCs could also be due to the fact that these cells were substantially involved in physiological bone remodeling, as also described in the literature (Hadjidakis and Androulakis, 2006). With TCP, many macrophages and some FBCs were present at each observation time point, and their amount was significantly higher than with the CMPCs at week 24. Therefore, it can be assumed that these two cell types were mainly responsible for the material degradation of TCP. Since the complete degradation of TCP happens rather slowly (Moore et al., 2001; Kolk et al., 2012; Bohner et al., 2020) and at the end of the study period larger amounts of potentially material-resorbing cells were still present, it can be assumed that the degradation of TCP was not yet completed after 24 weeks. However, as also described in the literature (Wiltfang et al., 2002), in the present study cellular degradation may have been impaired by the fact that a very high proportion of the numerous TCP fragments still present after 24 weeks were completely surrounded by the newly formed bone trabeculae and were therefore not accessible for further degradation for the time being. The final degradation of these particles occurs only when the material is exposed during remodeling processes of the newly formed bone (Wiltfang et al., 2002).

The resorption zone, found with an annular area of darker gray value within the scaffold volume of the CMPCs between weeks 2 and 8 in the *in-vivo* µCT scans, could be identified in the histological examination as fibrovascular, macrophage-rich stroma between the material core and the ingrowing bone trabeculae. Investigating brushite or K-struvite cements, other authors have also observed such a fibrovascular resorption zone around the cement during the early implantation period (up to 2 months) (Constantz et al., 1998; Frayssinet et al., 2000; Apelt et al., 2004; Kaiser et al., 2022). As this zone was significantly more frequent in Mg225d than in Mg225p and TCP, it is reasonable to assume a connection with the scaffold degradation rate. It is likely that the volume degradation in Mg225d and in some cases also in Mg225p proceeded too rapidly, rendering the attachment of the newly formed bone trabeculae to the scaffold material impossible, as also assumed for K-struvite by Kaiser et al. (2022). However, the fibrovascular zone formed to bridge the defect was in the present study at the latest after 10 weeks replaced by ingrowing trabecular bone.

Using SEM and EDX analysis, the surface texture of the thin sections of the scaffold-bone-complexes and the occurrence, respectively amount, of Mg in the implantation area have clearly demonstrated the presence of small scaffold particles in all materials by week 24. The results are consistent with the histological findings and confirm excellent osseointegration, almost complete degradation of the CMPCs, and replacement of the scaffolds by trabecular bone. In a study by Fuchs et al. (2021), EDX analysis of CMPCs granules also showed increasing degradation over time and their replacement by bone tissue based on the detection of Mg, Ca, and P. For TCP, very good osseointegration and osteoneogenesis were also observed in the present study, but only a slight progression of degradation occurred.

## 5 Conclusion

This study varied the post-treatment of 3D powder printed CMPC scaffolds by either immersion in DAHP or by infiltration with PA, and the influence of the post-treatment on the *in-vivo* performance of the scaffolds was examined. In a non-weight-bearing borehole defect in rabbits, both investigated CMPCs, Mg225d (alkaline post-treatment with DAHP) and Mg225p (acid post-treatment with PA), showed excellent biocompatibility and osseointegration, over time continuously increasing and almost complete degradation, and replacement of the scaffolds by newly formed bone trabeculae, which underwent continuous remodeling and adaption to the physiological situation. Post-treatment with DAHP resulted in significantly faster degradation with loss of cylindrical form, demarcability from surrounding bone, and scaffold volume in Mg225d than in Mg225p and TCP. Mg225d also showed significantly greater degradation on the side towards the medullary cavity than Mg225p and TCP. In TCP, degradation was significantly less than in the CMPCs after 24 weeks. All materials rapidly showed an ingrowth of numerous bone trabeculae into the scaffold. While in Mg225d, the trabeculae were predominantly located in the implantation area towards the cortex, in Mg225p they were more evenly distributed and showed almost the same structural properties as physiological bone at this localization after 24 weeks. The rapid degradation of Mg225d as well as the rapid breakdown of the scaffold framework into individual material particles probably had a negative effect on the uniform trabecular ingrowth. Therefore, and due to the low compressive strength of Mg225d, which presumably further decreased with increasing implantation time, this material is not suitable for application in weight-bearing bone. Since Mg225d nevertheless had a positive influence on osteoneogenesis, its use as a bone substitute in non-weight-bearing bone, such as for a sinus lift, would be feasible. In the present study, Mg225p showed, due to its higher compressive strength, optimal degradation rate for concurrent new bone formation, and excellent osteoneogenesis throughout the scaffold volume, promising properties for use as degradable bone substitute to be further investigated in weight-bearing bone.

## Data Availability

The original contributions presented in the study are included in the article/Supplementary Material, further inquiries can be directed to the corresponding author.

## References

[B1] Agarwal R. García A. J. (2015). Biomaterial strategies for engineering implants for enhanced osseointegration and bone repair. Adv. Drug Deliv. Rev. 94, 53–62. 10.1016/j.addr.2015.03.013 25861724 PMC4598264

[B2] Al-Maawi S. Barbeck M. Vizcaíno C. H. Egli R. Sader R. Kirkpatrick C. J. (2021). Thermal treatment at 500°C significantly reduces the reaction to irregular tricalcium phosphate granules as foreign bodies: An *in vivo* study. Acta Biomater. 121, 621–636. 10.1016/j.actbio.2020.11.034 33249227

[B3] Albrektsson T. Johansson C. (2001). Osteoinduction, osteoconduction and osseointegration. Eur. Spine J. 10 (2), 96–101. 10.1007/s005860100282 PMC3611551 11716023

[B4] Ambard A. J. Mueninghoff L. (2006). Calcium phosphate cement: Review of mechanical and biological properties. J. Prosthodont. 15 (5), 321–328. 10.1111/j.1532-849X.2006.00129.x 16958734

[B5] Apelt D. Theiss F. El-Warrak A. Zlinszky K. Bettschart-Wolfisberger R. Bohner M. (2004). *In vivo* behavior of three different injectable hydraulic calcium phosphate cements. Biomaterials 25 (7-8), 1439–1451. 10.1016/j.biomaterials.2003.08.073 14643619

[B6] Arrington E. D. Smith W. J. Chambers H. G. Bucknell A. L. Davino N. A. (1996). Complications of iliac crest bone graft harvesting. Clin. Orthop. Relat. Res. 329, 300–309. 10.1097/00003086-199608000-00037 8769465

[B7] Augustin J. Feichtner F. Waselau A. C. Julmi S. Klose C. Wriggers P. (2020). Comparison of two pore sizes of LAE442 scaffolds and their effect on degradation and osseointegration behavior in the rabbit model. J. Biomed. Mat. Res. 108 (7), 2776–2788. 10.1002/jbm.b.34607 32170913

[B8] Augustin J. Feichtner F. Waselau A. C. Julmi S. Klose C. Wriggers P. (2022). Effect of pore size on tissue ingrowth and osteoconductivity in biodegradable Mg alloy scaffolds. J. Appl. Biomater. Funct. Mat. 20, 228080002210781. 10.1177/22808000221078168 35189733

[B9] Bohner M. Santoni B. L. G. Döbelin N. (2020). β-tricalcium phosphate for bone substitution: Synthesis and properties. Acta Biomater. 113, 23–41. 10.1016/j.actbio.2020.06.022 32565369

[B10] Bohner M. Theiss F. Apelt D. Hirsiger W. Houriet R. Rizzoli G. (2003). Compositional changes of a dicalcium phosphate dihydrate cement after implantation in sheep. Biomaterials 24 (20), 3463–3474. 10.1016/s0142-9612(03)00234-5 12809775

[B11] Boyan B. D. Hummert T. W. Dean D. D. Schwartz Z. (1996). Role of material surfaces in regulating bone and cartilage cell response. Biomaterials 17 (2), 137–146. 10.1016/0142-9612(96)85758-9 8624390

[B12] Brunello G. Sivolella S. Meneghello R. Ferroni L. Gardin C. Piattelli A. (2016). Powder-based 3D printing for bone tissue engineering. Biotechnol. Adv. 34 (5), 740–753. 10.1016/j.biotechadv.2016.03.009 27086202

[B13] Campana V. Milano G. Pagano E. Barba M. Cicione C. Salonna G. (2014). Bone substitutes in orthopaedic surgery: From basic science to clinical practice. J. Mat. Sci. Mat. Med. 25 (10), 2445–2461. 10.1007/s10856-014-5240-2 PMC4169585 24865980

[B14] Castilho M. Dias M. Vorndran E. Gbureck U. Fernandes P. Pires I. (2014a). Application of a 3D printed customized implant for canine cruciate ligament treatment by tibial tuberosity advancement. Biofabrication 6 (2), 025005. 10.1088/1758-5082/6/2/025005 24658159

[B15] Castilho M. Moseke C. Ewald A. Gbureck U. Groll J. Pires I. (2014b). Direct 3D powder printing of biphasic calcium phosphate scaffolds for substitution of complex bone defects. Biofabrication 6 (1), 015006. 10.1088/1758-5082/6/1/015006 24429776

[B16] Chai Y. C. Carlier A. Bolander J. Roberts S. J. Geris L. Schrooten J. (2012). Current views on calcium phosphate osteogenicity and the translation into effective bone regeneration strategies. Acta Biomater. 8 (11), 3876–3887. 10.1016/j.actbio.2012.07.002 22796326

[B17] Chopra P. M. Johnson M. Nagy T. R. Lemons J. E. (2009). Micro-computed tomographic analysis of bone healing subsequent to graft placement. J. Biomed. Mat. Res. 88 (2), 611–618. 10.1002/jbm.b.31232 18837447

[B18] Constantz B. R. Barr B. M. Ison I. C. Fulmer M. T. Baker J. McKinney L. (1998). Histological, chemical, and crystallographic analysis of four calcium phosphate cements in different rabbit osseous sites. J. Biomed. Mat. Res. 43 (4), 451–461. 10.1002/(sici)1097-4636(199824)43:4<451:aid-jbm13>3.0.co;2-q 9855204

[B19] Donath K. Breuner G. (1982). A method for the study of undecalcified bones and teeth with attached soft tissues. The Säge‐Schliff (sawing and grinding) Technique. J. Oral Pathol. Med. 11 (4), 318–326. 10.1111/j.1600-0714.1982.tb00172.x 6809919

[B20] Dorozhkin S. V. (2008). Calcium orthophosphate cements for biomedical application. J. Mat. Sci. 43 (9), 3028–3057. 10.1007/s10853-008-2527-z

[B21] Dorozhkin S. V. (2013). Calcium orthophosphate-based bioceramics. Mater. (Basel) 6 (9), 3840–3942. 10.3390/ma6093840 PMC5452669 28788309

[B22] Dorozhkin S. V. Epple M. (2002). Biological and medical significance of calcium phosphates. Angew. Chem. Int. Ed. Engl. 41 (17), 3130–3146. 10.1002/1521-3773(20020902)41:17<3130:AID-ANIE3130>3.0.CO;2-1 12207375

[B23] Ewald A. Kreczy D. Brückner T. Gbureck U. Bengel M. Hoess A. (2019). Development and bone regeneration capacity of premixed magnesium phosphate cement pastes. Mater. (Basel) 12 (13), 2119. 10.3390/ma12132119 PMC6651064 31266228

[B24] Fillingham Y. Jacobs J. (2016). Bone grafts and their substitutes. Bone Jt. J. 98-b (1), 6–9. 10.1302/0301-620x.98b.36350 26733632

[B25] Frakenburg E. P. Goldstein S. A. Bauer T. W. Harris S. A. Poser R. D. (1998). Biomechanical and histological evaluation of a calcium phosphate cement. J. Bone Jt. Surg. 80 (8), 1112–1124. 10.2106/00004623-199808000-00004 9730120

[B26] Frayssinet P. Roudier M. Lerch A. Ceolin J. L. Deprès E. Rouquet N. (2000). Tissue reaction against a self-setting calcium phosphate cement set in bone or outside the organism. J. Mat. Sci. Mat. Med. 11 (12), 811–815. 10.1023/a:1008909714090 15348065

[B27] Fuchs A. Kreczy D. Brückner T. Gbureck U. Stahlhut P. Bengel M. (2021). Bone regeneration capacity of newly developed spherical magnesium phosphate cement granules. Clin. Oral Investig. 26, 2619–2633. 10.1007/s00784-021-04231-w PMC8898248 34686919

[B28] Gefel E. Moseke C. Schmitt A.-M. Dümmler N. Stahlhut P. Ewald A. (2022). Degradation of 3D-printed magnesium phosphate ceramics *in vitro* and a prognosis on their bone regeneration potential. Bioact. Mat. 19, 376–391. 10.1016/j.bioactmat.2022.04.015 PMC9127104 35633870

[B29] Ghanaati S. Barbeck M. Orth C. Willershausen I. Thimm B. W. Hoffmann C. (2010). Influence of β-tricalcium phosphate granule size and morphology on tissue reaction *in vivo* . Acta Biomater. 6 (12), 4476–4487. 10.1016/j.actbio.2010.07.006 20624495

[B30] Götz L. M. Holeczek K. Groll J. Jüngst T. Gbureck U. (2021). Extrusion-based 3D printing of calcium magnesium phosphate cement pastes for degradable bone implants. Mater. (Basel) 14 (18), 5197. 10.3390/ma14185197 PMC8472049 34576421

[B31] Gross K. A. Berndt C. C. (2002). Biomedical application of apatites. Rev. Mineral. Geochem. 48 (1), 631–672. 10.2138/rmg.2002.48.17

[B32] Habibovic P. Gbureck U. Doillon C. J. Bassett D. C. van Blitterswijk C. A. Barralet J. E. (2008). Osteoconduction and osteoinduction of low-temperature 3D printed bioceramic implants. Biomaterials 29 (7), 944–953. 10.1016/j.biomaterials.2007.10.023 18055009

[B33] Hadjidakis D. J. Androulakis I. I. (2006). Bone remodeling. Ann. N. Y. Acad. Sci. 1092, 385–396. 10.1196/annals.1365.035 17308163

[B34] Haque M. A. Chen B. (2020). *In vitro* and *in vivo* research advancements on the magnesium phosphate cement biomaterials: A review. Materialia 13, 100852. 10.1016/j.mtla.2020.100852

[B35] He L. Zhang X. Liu B. Tian Y. Ma W. (2016). Effect of magnesium ion on human osteoblast activity. Braz. J. Med. Biol. Res. 49 (7), S0100879X2016000700604. 10.1590/1414-431X20165257 PMC4942226 27383121

[B36] Höh N. v. d. Bormann D. Lucas A. Denkena B. Hackenbroich C. Meyer‐Lindenberg A. (2009). Influence of different surface machining treatments of magnesium‐based resorbable implants on the degradation behavior in rabbits. Adv. Eng. Mat. 11 (5), B47–B54. 10.1002/adem.200800273

[B37] Horch H. H. Sader R. Pautke C. Neff A. Deppe H. Kolk A. (2006). Synthetic, pure-phase beta-tricalcium phosphate ceramic granules (Cerasorb) for bone regeneration in the reconstructive surgery of the jaws. Int. J. Oral Maxillofac. Surg. 35 (8), 708–713. 10.1016/j.ijom.2006.03.017 16690249

[B38] Horowitz M. C. Berry R. Holtrup B. Sebo Z. Nelson T. Fretz J. A. (2017). Bone marrow adipocytes. Adipocyte 6 (3), 193–204. 10.1080/21623945.2017.1367881 28872979 PMC5638373

[B39] Huber F. X. McArthur N. Heimann L. Dingeldein E. Cavey H. Palazzi X. (2009). Evaluation of a novel nanocrystalline hydroxyapatite paste Ostim in comparison to Alpha-BSM - more bone ingrowth inside the implanted material with Ostim compared to Alpha BSM. BMC Musculoskelet. Disord. 10, 164. 10.1186/1471-2474-10-164 20028538 PMC2807853

[B40] Huehnerschulte T. A. Reifenrath J. von Rechenberg B. Dziuba D. Seitz J. M. Bormann D. (2012). *In vivo* assessment of the host reactions to the biodegradation of the two novel magnesium alloys ZEK100 and AX30 in an animal model. Biomed. Eng. Online 11 (1), 14. 10.1186/1475-925X-11-14 22429539 PMC3352308

[B41] Jia J. Zhou H. Wei J. Jiang X. Hua H. Chen F. (2010). Development of magnesium calcium phosphate biocement for bone regeneration. J. R. Soc. Interface 7 (49), 1171–1180. 10.1098/rsif.2009.0559 20181560 PMC2894874

[B42] Kaiser F. Schröter L. Stein S. Krüger B. Weichhold J. Stahlhut P. (2022). Accelerated bone regeneration through rational design of magnesium phosphate cements. Acta Biomater. 145, 358–371. 10.1016/j.actbio.2022.04.019 35443213

[B43] Kanter B. Geffers M. Ignatius A. Gbureck U. (2014). Control of *in vivo* mineral bone cement degradation. Acta Biomater. 10 (7), 3279–3287. 10.1016/j.actbio.2014.04.020 24769112

[B44] Kanter B. (2014). Osseointegration kalthärtender Knochenzemente im Schafmodell. Ludwig-Maximilians-University Munich. dissertation. Munich.

[B45] Kanter B. Vikman A. Brückner T. Schamel M. Gbureck U. Ignatius A. (2018). Bone regeneration capacity of magnesium phosphate cements in a large animal model. Acta Biomater. 69, 352–361. 10.1016/j.actbio.2018.01.035 29409867

[B46] Karageorgiou V. Kaplan D. (2005). Porosity of 3D biomaterial scaffolds and osteogenesis. Biomaterials 26 (27), 5474–5491. 10.1016/j.biomaterials.2005.02.002 15860204

[B47] Kasuya A. Sobajima S. Kinoshita M. (2012). *In vivo* degradation and new bone formation of calcium phosphate cement-gelatin powder composite related to macroporosity after *in situ* gelatin degradation. J. Orthop. Res. 30 (7), 1103–1111. 10.1002/jor.22044 22213166

[B48] Katsimbri P. (2017). The biology of normal bone remodelling. Eur. J. Cancer Care 26 (6), e12740. 10.1111/ecc.12740 28786518

[B49] Keating J. F. McQueen M. M. (2001). Substitutes for autologous bone graft in orthopaedic trauma. J. Bone Jt. Surg. Br. volume 83 (1), 3–8. 10.1302/0301-620x.83b1.0830003 11245534

[B50] Kheirallah M. Almeshaly H. (2016). Bone graft substitutes for bone defect regeneration. A collective review. Int. J. Dent. Oral Sci. 3 (5), 247–255. 10.19070/2377-8075-1600051

[B51] Kim J.-A. Lim J. Naren R. Yun H.-S. Park E. K. (2016). Effect of the biodegradation rate controlled by pore structures in magnesium phosphate ceramic scaffolds on bone tissue regeneration *in vivo* . Acta Biomater. 44, 155–167. 10.1016/j.actbio.2016.08.039 27554019

[B52] Klammert U. Ignatius A. Wolfram U. Reuther T. Gbureck U. (2011). *In vivo* degradation of low temperature calcium and magnesium phosphate ceramics in a heterotopic model. Acta Biomater. 7 (9), 3469–3475. 10.1016/j.actbio.2011.05.022 21658480

[B53] Klammert U. Reuther T. Blank M. Reske I. Barralet J. E. Grover L. M. (2010a). Phase composition, mechanical performance and *in vitro* biocompatibility of hydraulic setting calcium magnesium phosphate cement. Acta Biomater. 6 (4), 1529–1535. 10.1016/j.actbio.2009.10.021 19837194

[B54] Klammert U. Vorndran E. Reuther T. Müller F. A. Zorn K. Gbureck U. (2010b). Low temperature fabrication of magnesium phosphate cement scaffolds by 3D powder printing. J. Mat. Sci. Mat. Med. 21 (11), 2947–2953. 10.1007/s10856-010-4148-8 20740307

[B55] Kleer N. Julmi S. Gartzke A.-K. Augustin J. Feichtner F. Waselau A.-C. (2019). Comparison of degradation behaviour and osseointegration of the two magnesium scaffolds, LAE442 and La2, *in vivo* . Materialia 8, 100436. 10.1016/j.mtla.2019.100436

[B56] Kleer-Reiter N. Julmi S. Feichtner F. Waselau A. C. Klose C. Wriggers P. (2021). Biocompatibility and degradation of the open-pored magnesium scaffolds LAE442 and La2. Biomed. Mat. 16 (3), 035037. 10.1088/1748-605X/abf5c5 33827052

[B57] Kolk A. Handschel J. Drescher W. Rothamel D. Kloss F. Blessmann M. (2012). Current trends and future perspectives of bone substitute materials–from space holders to innovative biomaterials. J. Cranio-Maxillofacial Surg. 40 (8), 706–718. 10.1016/j.jcms.2012.01.002 22297272

[B58] Kowalewicz K. Vorndran E. Feichtner F. Waselau A.-C. Brueckner M. Meyer-Lindenberg A. (2021). *In-vivo* degradation behavior and osseointegration of 3D powder-printed calcium magnesium phosphate cement scaffolds. Mater. (Basel) 14 (4), 946. 10.3390/ma14040946 PMC7923127 33671265

[B59] Kurashina K. Kurita H. Kotani A. Takeuchi H. Hirano M. (1997). *In vivo* study of a calcium phosphate cement consisting of α-tricalcium phosphate/dicalcium phosphate dibasic/tetracalcium phosphate monoxide. Biomaterials 18 (2), 147–151. 10.1016/s0142-9612(96)00173-1 9022962

[B60] Laurie S. W. Kaban L. B. Mulliken J. B. Murray J. E. (1984). Donor-site morbidity after harvesting rib and iliac bone. Plastic Reconstr. Surg. 73 (6), 933–938. 10.1097/00006534-198406000-00014 6374708

[B61] LeGeros R. Z. (2008). Calcium phosphate-based osteoinductive materials. Chem. Rev. 108 (11), 4742–4753. 10.1021/cr800427g 19006399

[B62] LeGeros R. Z. (2002). Properties of osteoconductive biomaterials: Calcium phosphates. Clin. Orthop. Relat. Res. 395, 81–98. 10.1097/00003086-200202000-00009 11937868

[B63] Lodoso-Torrecilla I. van den Beucken J. Jansen J. A. (2021). Calcium phosphate cements: Optimization toward biodegradability. Acta Biomater. 119, 1–12. 10.1016/j.actbio.2020.10.013 33065287

[B64] Lucas D. (2021). Structural organization of the bone marrow and its role in hematopoiesis. Curr. Opin. Hematol. 28 (1), 36–42. 10.1097/moh.0000000000000621 33177411 PMC7769132

[B65] Mestres G. Ginebra M.-P. (2011). Novel magnesium phosphate cements with high early strength and antibacterial properties. Acta Biomater. 7 (4), 1853–1861. 10.1016/j.actbio.2010.12.008 21147277

[B66] Moore W. R. Graves S. E. Bain G. I. (2001). Synthetic bone graft substitutes. ANZ J. Surg. 71 (6), 354–361. 10.1046/j.1440-1622.2001.02128.x 11409021

[B67] Nabiyouni M. Brückner T. Zhou H. Gbureck U. Bhaduri S. B. (2018). Magnesium-based bioceramics in orthopedic applications. Acta Biomater. 66, 23–43. 10.1016/j.actbio.2017.11.033 29197578

[B68] Nombela-Arrieta C. Manz M. G. (2017). Quantification and three-dimensional microanatomical organization of the bone marrow. Blood Adv. 1 (6), 407–416. 10.1182/bloodadvances.2016003194 29296956 PMC5738992

[B69] Ostrowski N. Roy A. Kumta P. N. (2016). Magnesium phosphate cement systems for hard tissue applications: A review. ACS Biomater. Sci. Eng. 2 (7), 1067–1083. 10.1021/acsbiomaterials.6b00056 33445235

[B70] Peters F. Groisman D. Davids R. Hänel T. Dürr H. Klein M. (2006). Comparative study of patient individual implants from β‐tricalcium phosphate made by different techniques based on CT data. Materwiss. Werksttech. 37 (6), 457–461. 10.1002/mawe.200600019

[B71] Reid J. W. Hendry J. A. (2006). Rapid, accurate phase quantification of multiphase calcium phosphate materials using Rietveld refinement. J. Appl. Cryst. 39 (4), 536–543. 10.1107/S0021889806020395

[B72] Rentsch C. Rentsch B. Scharnweber D. Zwipp H. Rammelt S. (2012). [Bone substitute. Transplants and replacement materials-an update]. Knochenersatz. Unfallchirurg 115 (10), 938–949. 10.1007/s00113-012-2238-4 22821191

[B73] Rietveld H. M. (1969). A profile refinement method for nuclear and magnetic structures. J. Appl. Cryst. 2 (2), 65–71. 10.1107/S0021889869006558

[B74] Roseti L. Parisi V. Petretta M. Cavallo C. Desando G. Bartolotti I. (2017). Scaffolds for bone tissue engineering: State of the art and new perspectives. Mater. Sci. Eng. C 78, 1246–1262. 10.1016/j.msec.2017.05.017 28575964

[B75] Samavedi S. Whittington A. R. Goldstein A. S. (2013). Calcium phosphate ceramics in bone tissue engineering: A review of properties and their influence on cell behavior. Acta Biomater. 9 (9), 8037–8045. 10.1016/j.actbio.2013.06.014 23791671

[B76] Sarkar K. Kumar V. Devi K. B. Ghosh D. Nandi S. K. Roy M. (2019). Effects of Sr doping on biodegradation and bone regeneration of magnesium phosphate bioceramics. Materialia 5, 100211. 10.1016/j.mtla.2019.100211

[B77] Theiss F. Apelt D. Brand B. Kutter A. Zlinszky K. Bohner M. (2005). Biocompatibility and resorption of a brushite calcium phosphate cement. Biomaterials 26 (21), 4383–4394. 10.1016/j.biomaterials.2004.11.056 15701367

[B78] Travlos G. S. (2006). Normal structure, function, and histology of the bone marrow. Toxicol. Pathol. 34 (5), 548–565. 10.1080/01926230600939856 17067943

[B79] von Doernberg M.-C. von Rechenberg B. Bohner M. Grünenfelder S. van Lenthe G. H. Müller R. (2006). *In vivo* behavior of calcium phosphate scaffolds with four different pore sizes. Biomaterials 27 (30), 5186–5198. 10.1016/j.biomaterials.2006.05.051 16790273

[B80] Vorndran E. Ewald A. Müller F. A. Zorn K. Kufner A. Gbureck U. (2011). Formation and properties of magnesium-ammonium-phosphate hexahydrate biocements in the Ca-Mg-PO4 system. J. Mat. Sci. Mat. Med. 22 (3), 429–436. 10.1007/s10856-010-4220-4 21221732

[B81] Vorndran E. Klarner M. Klammert U. Grover L. M. Patel S. Barralet J. E. (2008). 3D powder printing of β‐tricalcium phosphate ceramics using different strategies. Adv. Eng. Mat. 10 (12), B67–B71. 10.1002/adem.200800179

[B82] Walsh W. R. Vizesi F. Michael D. Auld J. Langdown A. Oliver R. (2008). β-TCP bone graft substitutes in a bilateral rabbit tibial defect model. Biomaterials 29 (3), 266–271. 10.1016/j.biomaterials.2007.09.035 18029011

[B83] Wang L. Nancollas G. H. (2008). Calcium orthophosphates: Crystallization and dissolution. Chem. Rev. 108 (11), 4628–4669. 10.1021/cr0782574 18816145 PMC2743557

[B84] Wang S. Xu C. Yu S. Wu X. Jie Z. Dai H. (2019). Citric acid enhances the physical properties, cytocompatibility and osteogenesis of magnesium calcium phosphate cement. J. Mech. Behav. Biomed. Mat. 94, 42–50. 10.1016/j.jmbbm.2019.02.026 30856478

[B85] Wei J. Jia J. Wu F. Wei S. Zhou H. Zhang H. (2010). Hierarchically microporous/macroporous scaffold of magnesium–calcium phosphate for bone tissue regeneration. Biomaterials 31 (6), 1260–1269. 10.1016/j.biomaterials.2009.11.005 19931903

[B86] Willbold E. Witte F. (2010). Histology and research at the hard tissue–implant interface using Technovit 9100 New embedding technique. Acta Biomater. 6 (11), 4447–4455. 10.1016/j.actbio.2010.06.022 20601246

[B87] Wiltfang J. Merten H. A. Schlegel K. A. Schultze-Mosgau S. Kloss F. R. Rupprecht S. (2002). Degradation characteristics of alpha and beta tri-calcium-phosphate (TCP) in minipigs. J. Biomed. Mat. Res. 63 (2), 115–121. 10.1002/jbm.10084 11870643

[B88] Wu F. Su J. Wei J. Guo H. Liu C. (2008a). Injectable bioactive calcium–magnesium phosphate cement for bone regeneration. Biomed. Mat. 3 (4), 044105. 10.1088/1748-6041/3/4/044105 19029607

[B89] Wu F. Wei J. Guo H. Chen F. Hong H. Liu C. (2008b). Self-setting bioactive calcium–magnesium phosphate cement with high strength and degradability for bone regeneration. Acta Biomater. 4 (6), 1873–1884. 10.1016/j.actbio.2008.06.020 18662897

[B90] Wu L. Feyerabend F. Schilling A. F. Willumeit-Römer R. Luthringer B. J. (2015). Effects of extracellular magnesium extract on the proliferation and differentiation of human osteoblasts and osteoclasts in coculture. Acta Biomater. 27, 294–304. 10.1016/j.actbio.2015.08.042 26318802

[B91] Xu L. Yu G. Zhang E. Pan F. Yang K. (2007). *In vivo* corrosion behavior of Mg‐Mn‐Zn alloy for bone implant application. J. Biomed. Mat. Res. A 83 (3), 703–711. 10.1002/jbm.a.31273 17549695

[B92] Zeng D. Xia L. Zhang W. Huang H. Wei B. Huang Q. (2012). Maxillary sinus floor elevation using a tissue-engineered bone with calcium-magnesium phosphate cement and bone marrow stromal cells in rabbits. Tissue Eng. Part A 18 (7-8), 870–881. 10.1089/ten.TEA.2011.0379 22066969

[B93] Zhang E. Xu L. Yu G. Pan F. Yang K. (2009). *In vivo* evaluation of biodegradable magnesium alloy bone implant in the first 6 months implantation. J. Biomed. Mat. Res. A 90 (3), 882–893. 10.1002/jbm.a.32132 18618719

[B94] Zhang J. Liu W. Schnitzler V. Tancret F. Bouler J. M. (2014). Calcium phosphate cements for bone substitution: Chemistry, handling and mechanical properties. Acta Biomater. 10 (3), 1035–1049. 10.1016/j.actbio.2013.11.001 24231047

[B95] Zhang L. Yang G. Johnson B. N. Jia X. (2019). Three-dimensional (3D) printed scaffold and material selection for bone repair. Acta Biomater. 84, 16–33. 10.1016/j.actbio.2018.11.039 30481607

[B96] Zimmermann G. Moghaddam A. (2011). Allograft bone matrix versus synthetic bone graft substitutes. Injury 42, 16–21. 10.1016/j.injury.2011.06.199 21889142

