# Supplementary material for: Comparison of degradation behavior and osseointegration of 3D powder-printed calcium magnesium phosphate cement scaffolds with alkaline or acid post-treatment
Source: Front Bioeng Biotechnol. 2022 Sep 28;10:998254. doi: 10.3389/fbioe.2022.998254 (PMC9554004; doi:10.3389/fbioe.2022.998254)
Supplement: Supplementary file 1 [file DataSheet1.docx]

Supplementary Material

# Supplementary Tables

**Supplementary Table S1**. Semi-quantitative in-vivo µCT evaluation: Scaffold demarcability. Percentage of Mg225d, Mg225p and TCP scaffolds classified in scores 0, 1 and 2 over the study period (directly after surgery up to 24 weeks).

| **Scaffold demarcability** | | **Score** | | | |
| --- | --- | --- | --- | --- | --- |
| **Week** | **Material** | **0** | | **1** | **2** |
| 0 | Mg225d | | 0.00% | 0.00% | 100.00% |
|  | Mg225p | | 0.00% | 0.00% | 100.00% |
|  | TCP | | 0.00% | 0.00% | 100.00% |
| 2 | Mg225d | | 0.00% | 14.58% | 85.42% |
|  | Mg225p | | 0.00% | 2.08% | 97.92% |
|  | TCP | | 0.00% | 0.00% | 100.00% |
| 4 | Mg225d | | 0.00% | 58.70% | 41.30% |
|  | Mg225p | | 0.00% | 14.58% | 85.42% |
|  | TCP | | 0.00% | 0.00% | 100.00% |
| 6 | Mg225d | | 0.00% | 100.00% | 0.00% |
|  | Mg225p | | 0.00% | 70.83% | 29.17% |
|  | TCP | | 0.00% | 0.00% | 100.00% |
| 8 | Mg225d | | 31.25% | 68.75% | 0.00% |
|  | Mg225p | | 0.00% | 87.50% | 12.50% |
|  | TCP | | 0.00% | 0.00% | 100.00% |
| 10 | Mg225d | | 71.88% | 28.13% | 0.00% |
|  | Mg225p | | 3.13% | 96.88% | 0.00% |
|  | TCP | | 0.00% | 0.00% | 100.00% |
| 12 | Mg225d | | 90.63% | 9.38% | 0.00% |
|  | Mg225p | | 6.25% | 93.75% | 0.00% |
|  | TCP | | 0.00% | 0.00% | 100.00% |
| 16 | Mg225d | | 100.00% | 0.00% | 0.00% |
|  | Mg225p | | 31.25% | 68.75% | 0.00% |
|  | TCP | | 0.00% | 0.00% | 100.00% |
| 20 | Mg225d | | 100.00% | 0.00% | 0.00% |
|  | Mg225p | | 50.00% | 50.00% | 0.00% |
|  | TCP | | 0.00% | 0.00% | 100.00% |
| 24 | Mg225d | | 100.00% | 0.00% | 0.00% |
|  | Mg225p | | 56.25% | 43.75% | 0.00% |
|  | TCP | | 0.00% | 0.00% | 100.00% |

**Supplementary Table S2.** Semi-quantitative in-vivo µCT evaluation: Scaffold degradation. Percentage of Mg225d, Mg225p and TCP scaffolds classified in scores 0, 1 and 2 over the study period (directly after surgery up to 24 weeks). Due to the lack of demarcability of all Mg225d scaffolds from the bone tissue from week 16 onwards, the evaluation of scaffold degradation was no longer possible at weeks 16, 20 and 24.

| **Scaffold degradation** | | **Score** | | |
| --- | --- | --- | --- | --- |
| **Week** | **Material** | **0** | **1** | **2** |
| 0 | Mg225d | 100.00% | 0.00% | 0.00% |
|  | Mg225p | 100.00% | 0.00% | 0.00% |
|  | TCP | 100.00% | 0.00% | 0.00% |
| 2 | Mg225d | 81.25% | 18.75% | 0.00% |
|  | Mg225p | 100.00% | 0.00% | 0.00% |
|  | TCP | 100.00% | 0.00% | 0.00% |
| 4 | Mg225d | 43.48% | 56.52% | 0.00% |
|  | Mg225p | 91.67% | 8.33% | 0.00% |
|  | TCP | 100.00% | 0.00% | 0.00% |
| 6 | Mg225d | 19.15% | 72.34% | 8.51% |
|  | Mg225p | 85.42% | 14.58% | 0.00% |
|  | TCP | 97.92% | 2.08% | 0.00% |
| 8 | Mg225d | 9.09% | 50.00% | 40.91% |
|  | Mg225p | 71.88% | 21.88% | 6.25% |
|  | TCP | 84.38% | 15.63% | 0.00% |
| 10 | Mg225d | 22.22% | 0.00% | 77.78% |
|  | Mg225p | 61.29% | 32.26% | 6.45% |
|  | TCP | 56.25% | 43.75% | 0.00% |
| 12 | Mg225d | 33.33% | 0.00% | 66.67% |
|  | Mg225p | 70.00% | 23.33% | 6.67% |
|  | TCP | 50.00% | 50.00% | 0.00% |
| 16 | Mg225d | N/A | N/A | N/A |
|  | Mg225p | 81.82% | 18.18% | 0.00% |
|  | TCP | 31.25% | 68.75% | 0.00% |
| 20 | Mg225d | N/A | N/A | N/A |
|  | Mg225p | 62.50% | 37.50% | 0.00% |
|  | TCP | 43.75% | 56.25% | 0.00% |
| 24 | Mg225d | N/A | N/A | N/A |
|  | Mg225p | 66.67% | 33.33% | 0.00% |
|  | TCP | 43.75% | 56.25% | 0.00% |

**Supplementary Table S3.** Semi-quantitative in-vivo µCT evaluation: Scaffold form. Percentage of Mg225d, Mg225p and TCP scaffolds classified in scores 0, 1 and 2 over the study period (directly after surgery up to 24 weeks). Due to the lack of demarcability of all Mg225d scaffolds from the bone tissue from week 16 onwards, the evaluation of scaffold form was no longer possible at weeks 16, 20 and 24.

| **Scaffold form** | | **Score** | | |
| --- | --- | --- | --- | --- |
| **Week** | **Material** | **0** | **1** | **2** |
| 0 | Mg225d | 0.00% | 0.00% | 100.00% |
|  | Mg225p | 0.00% | 0.00% | 100.00% |
|  | TCP | 0.00% | 0.00% | 100.00% |
| 2 | Mg225d | 0.00% | 2.08% | 97.92% |
|  | Mg225p | 0.00% | 4.17% | 95.83% |
|  | TCP | 0.00% | 0.00% | 100.00% |
| 4 | Mg225d | 0.00% | 30.43% | 69.57% |
|  | Mg225p | 0.00% | 27.08% | 72.92% |
|  | TCP | 0.00% | 0.00% | 100.00% |
| 6 | Mg225d | 29.17% | 66.67% | 4.17% |
|  | Mg225p | 8.33% | 41.67% | 50.00% |
|  | TCP | 0.00% | 0.00% | 100.00% |
| 8 | Mg225d | 93.75% | 6.25% | 0.00% |
|  | Mg225p | 12.50% | 81.25% | 6.25% |
|  | TCP | 0.00% | 0.00% | 100.00% |
| 10 | Mg225d | 100.00% | 0.00% | 0.00% |
|  | Mg225p | 43.75% | 53.13% | 3.13% |
|  | TCP | 0.00% | 0.00% | 100.00% |
| 12 | Mg225d | 100.00% | 0.00% | 0.00% |
|  | Mg225p | 68.75% | 31.25% | 0.00% |
|  | TCP | 0.00% | 0.00% | 100.00% |
| 16 | Mg225d | N/A | N/A | N/A |
|  | Mg225p | 75.00% | 25.00% | 0.00% |
|  | TCP | 0.00% | 0.00% | 100.00% |
| 20 | Mg225d | N/A | N/A | N/A |
|  | Mg225p | 87.50% | 12.50% | 0.00% |
|  | TCP | 0.00% | 6.25% | 93.75% |
| 24 | Mg225d | N/A | N/A | N/A |
|  | Mg225p | 87.50% | 12.50% | 0.00% |
|  | TCP | 0.00% | 6.25% | 93.75% |

**Supplementary Table S4.** Semi-quantitative in-vivo µCT evaluation: Resorption zone. Percentage of Mg225d, Mg225p and TCP scaffolds classified in scores 0, 1 and 2 over the study period (directly after surgery up to 24 weeks). Due to the lack of demarcability of all Mg225d scaffolds from the bone tissue from week 16 onwards, the evaluation of a resorption zone was no longer possible at weeks 16, 20 and 24.

| **Resorption zone** | | **Score** | | |
| --- | --- | --- | --- | --- |
| **Week** | **Material** | **0** | **1** | **2** |
| 0 | Mg225d | 100.00% | 0.00% | 0.00% |
|  | Mg225p | 100.00% | 0.00% | 0.00% |
|  | TCP | 100.00% | 0.00% | 0.00% |
| 2 | Mg225d | 12.50% | 43.75% | 43.75% |
|  | Mg225p | 81.25% | 18.75% | 0.00% |
|  | TCP | 100.00% | 0.00% | 0.00% |
| 4 | Mg225d | 13.04% | 19.57% | 67.39% |
|  | Mg225p | 54.17% | 27.08% | 18.75% |
|  | TCP | 100.00% | 0.00% | 0.00% |
| 6 | Mg225d | 64.58% | 29.17% | 6.25% |
|  | Mg225p | 77.08% | 20.83% | 2.08% |
|  | TCP | 100.00% | 0.00% | 0.00% |
| 8 | Mg225d | 93.75% | 6.25% | 0.00% |
|  | Mg225p | 96.88% | 3.13% | 0.00% |
|  | TCP | 100.00% | 0.00% | 0.00% |
| 10 | Mg225d | 100.00% | 0.00% | 0.00% |
|  | Mg225p | 100.00% | 0.00% | 0.00% |
|  | TCP | 100.00% | 0.00% | 0.00% |
| 12 | Mg225d | 100.00% | 0.00% | 0.00% |
|  | Mg225p | 100.00% | 0.00% | 0.00% |
|  | TCP | 100.00% | 0.00% | 0.00% |
| 16 | Mg225d | N/A | N/A | N/A |
|  | Mg225p | 100.00% | 0.00% | 0.00% |
|  | TCP | 100.00% | 0.00% | 0.00% |
| 20 | Mg225d | N/A | N/A | N/A |
|  | Mg225p | 100.00% | 0.00% | 0.00% |
|  | TCP | 87.50% | 12.50% | 0.00% |
| 24 | Mg225d | N/A | N/A | N/A |
|  | Mg225p | 100.00% | 0.00% | 0.00% |
|  | TCP | 87.50% | 12.50% | 0.00% |

**Supplementary Table S5.** Semi-quantitative in-vivo µCT evaluation: Scaffold – bone – contact. Percentage of Mg225d, Mg225p and TCP scaffolds classified in scores 0, 1 and 2 over the study period (directly after surgery up to 24 weeks).

| **Scaffold – bone – contact** | | **Score** | | | |
| --- | --- | --- | --- | --- | --- |
| **Week** | **Material** | **0** | | **1** | **2** |
| 0 | Mg225d | | 0.00% | 0.00% | 100.00% |
|  | Mg225p | | 0.00% | 0.00% | 100.00% |
|  | TCP | | 0.00% | 0.00% | 100.00% |
| 2 | Mg225d | | 12.50% | 87.50% | 0.00% |
|  | Mg225p | | 14.58% | 83.33% | 2.08% |
|  | TCP | | 0.00% | 97.92% | 2.08% |
| 4 | Mg225d | | 89.13% | 10.87% | 0.00% |
|  | Mg225p | | 95.83% | 4.17% | 0.00% |
|  | TCP | | 93.48% | 6.52% | 0.00% |
| 6 | Mg225d | | 100.00% | 0.00% | 0.00% |
|  | Mg225p | | 97.92% | 2.08% | 0.00% |
|  | TCP | | 97.92% | 2.08% | 0.00% |
| 8 | Mg225d | | 100.00% | 0.00% | 0.00% |
|  | Mg225p | | 100.00% | 0.00% | 0.00% |
|  | TCP | | 100.00% | 0.00% | 0.00% |
| 10 | Mg225d | | 100.00% | 0.00% | 0.00% |
|  | Mg225p | | 100.00% | 0.00% | 0.00% |
|  | TCP | | 100.00% | 0.00% | 0.00% |
| 12 | Mg225d | | 100.00% | 0.00% | 0.00% |
|  | Mg225p | | 100.00% | 0.00% | 0.00% |
|  | TCP | | 100.00% | 0.00% | 0.00% |
| 16 | Mg225d | | 100.00% | 0.00% | 0.00% |
|  | Mg225p | | 100.00% | 0.00% | 0.00% |
|  | TCP | | 100.00% | 0.00% | 0.00% |
| 20 | Mg225d | | 100.00% | 0.00% | 0.00% |
|  | Mg225p | | 100.00% | 0.00% | 0.00% |
|  | TCP | | 100.00% | 0.00% | 0.00% |
| 24 | Mg225d | | 100.00% | 0.00% | 0.00% |
|  | Mg225p | | 100.00% | 0.00% | 0.00% |
|  | TCP | | 100.00% | 0.00% | 0.00% |

# Supplementary Figures


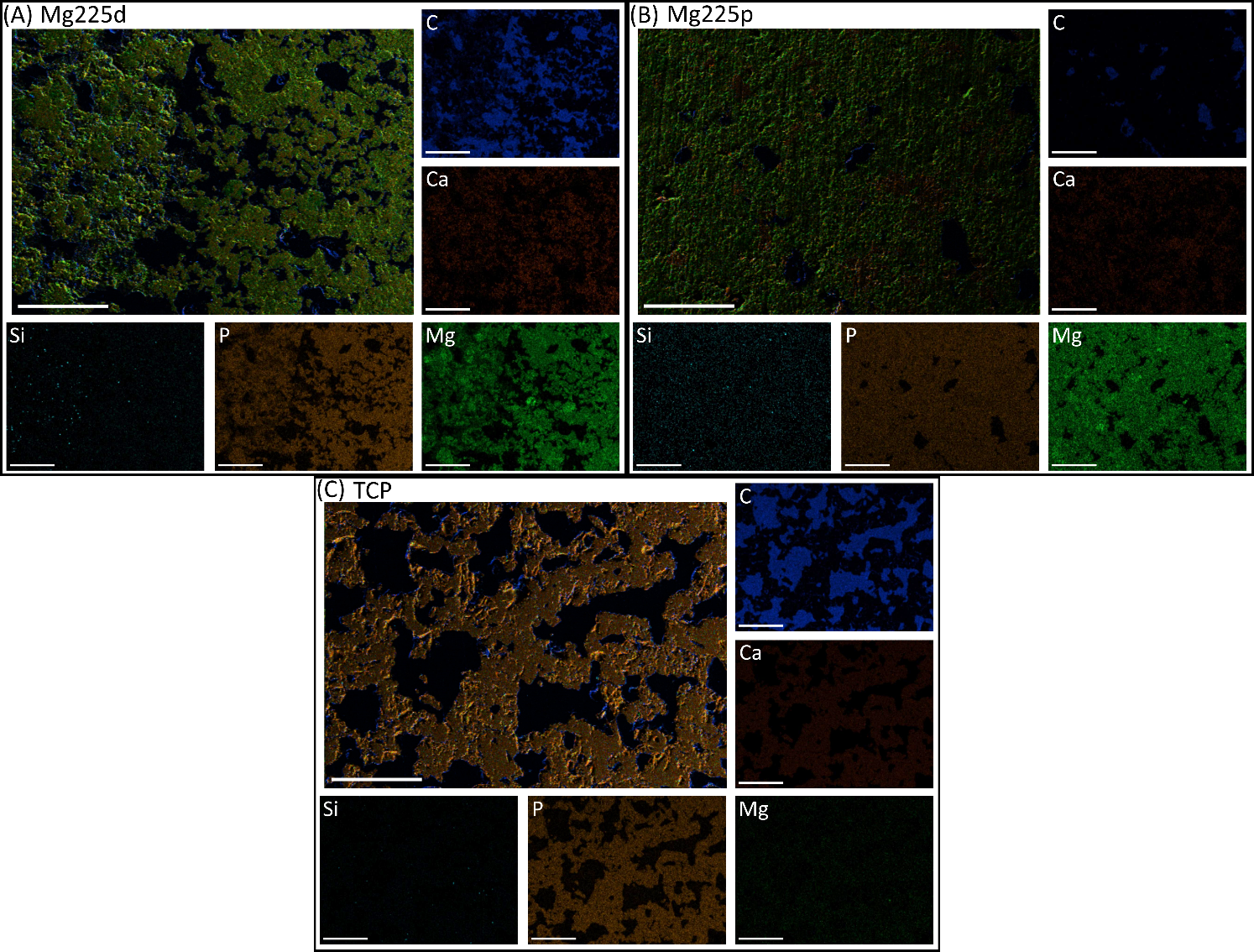


**Supplementary Figure S1.** EDX analysis (×100 magnification) from the scaffold center of (**A**) Mg225d, (**B**) Mg225p and (**C**) TCP prior to implantation. Scale bar = 250 µm.


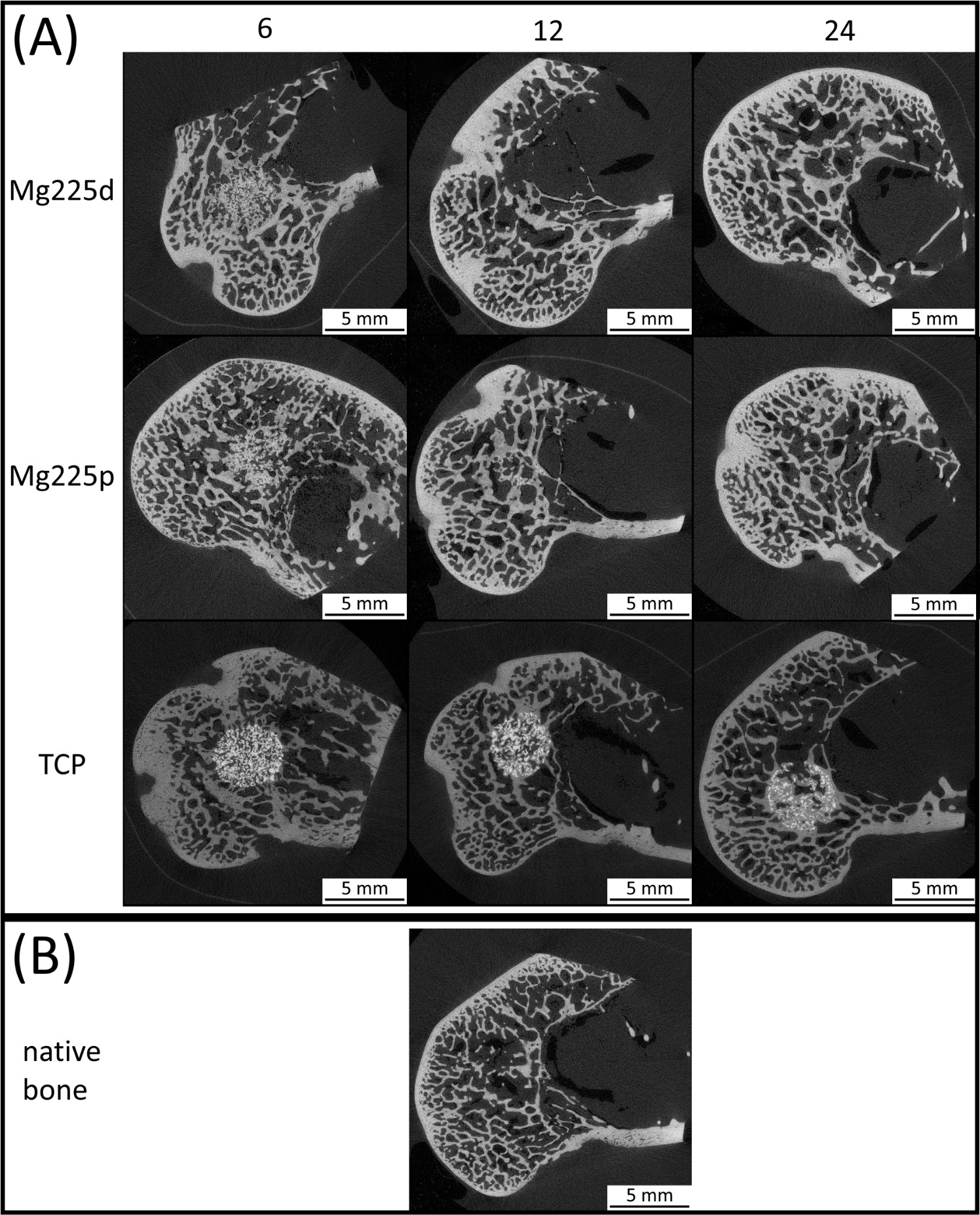


**Supplementary Figure S2.** Cross-sectional µCT 80 images (**A**) of the scaffolds (Mg225d, Mg225p and TCP) implanted in the distal femoral condyles at 6, 12 and 24 weeks after surgery (**B**) compared to native cancellous bone of the lateral femoral condyle.

**
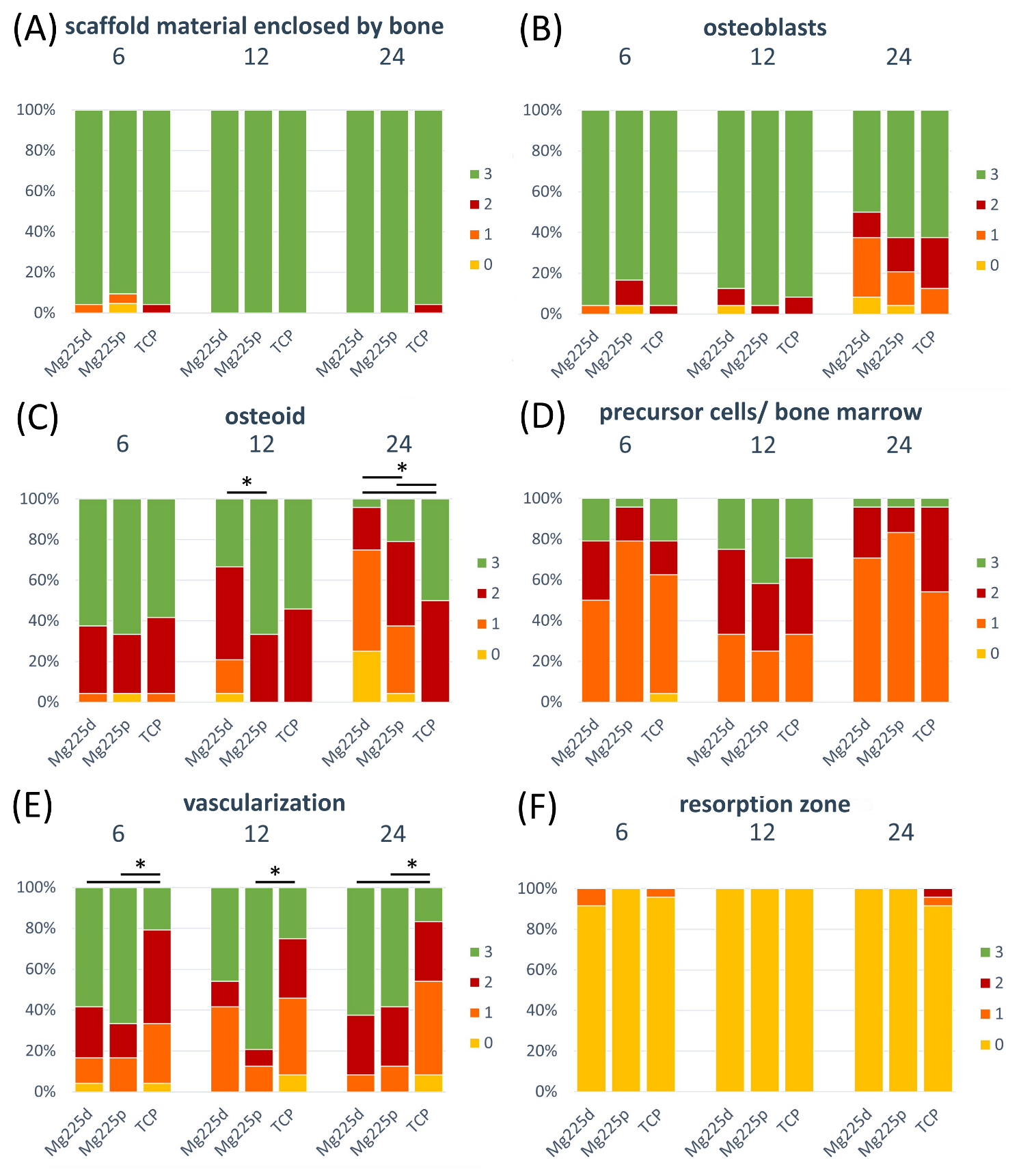
**

**Supplementary Figure S3**. Semi-quantitative histological evaluation of the scaffolds and the ingrowing tissue after 6, 12 and 24 weeks. *: Significant differences (p < 0.05) between the individual materials.

**
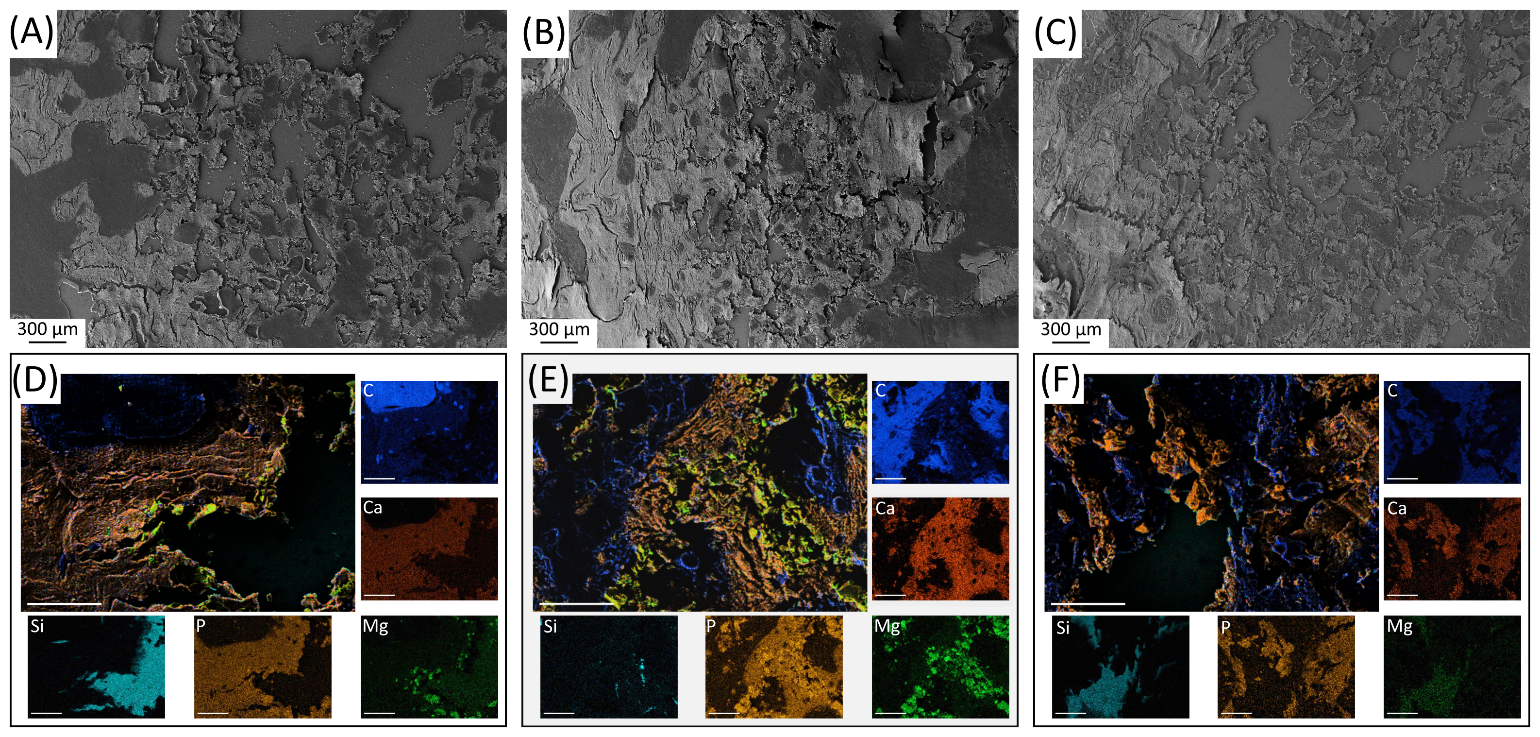
**

**Supplementary Figure S4**. (**A-C**) SEM analysis of histological thin sections (×28 magnification) with (**A**) Mg225d, (**B**) Mg225p and (**C**) TCP scaffold incorporated by newly formed cancellous bone after 6 weeks. (**D-F**) EDX analysis of histological thin sections (×500 magnification) with (**D**) Mg225d, (**E**) Mg225p and (**F**) TCP particles incorporated by newly formed cancellous bone after 6 weeks. Scale bar = 50 µm.


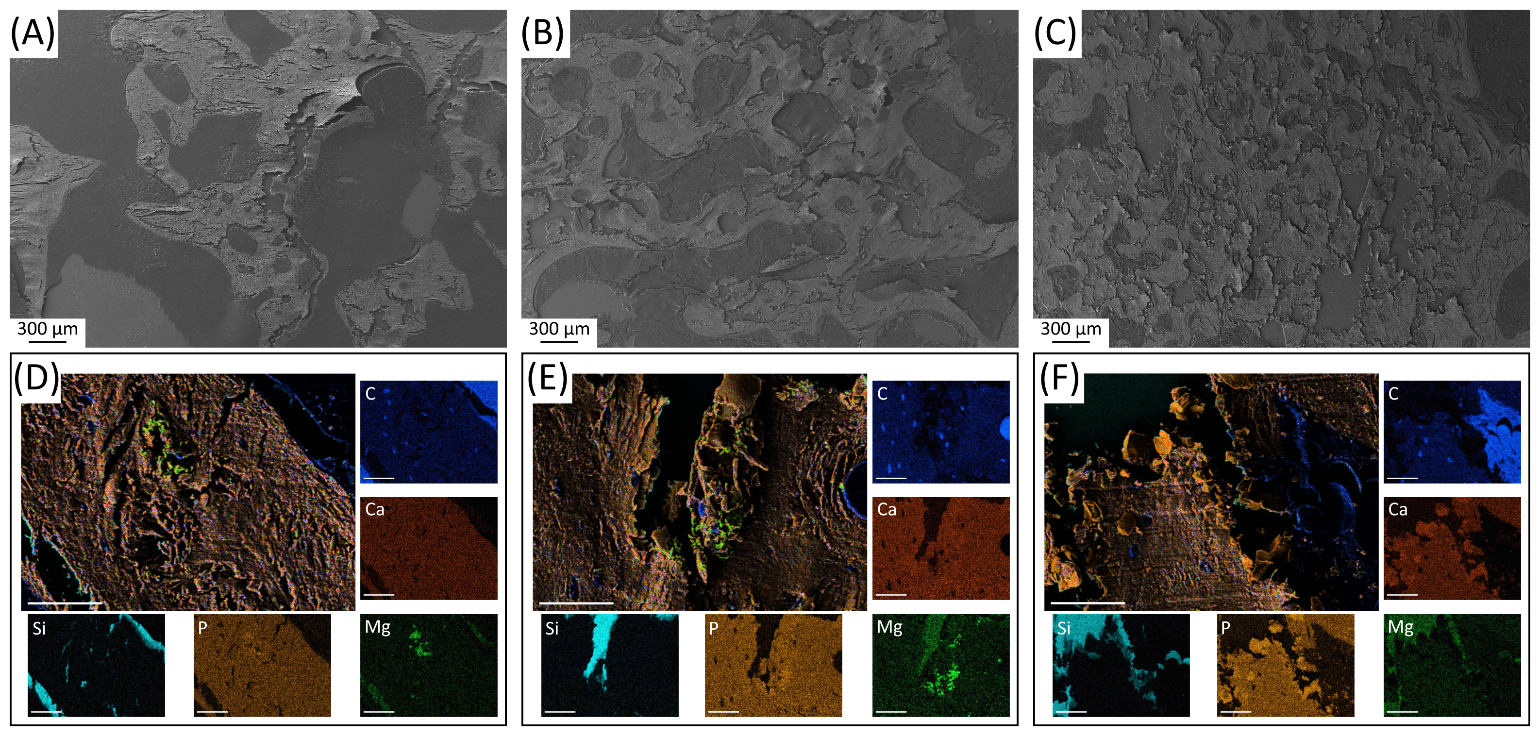


**Supplementary Figure S5**. (**A-C**) SEM analysis of histological thin sections (×28 magnification) with (**A**) Mg225d, (**B**) Mg225p and (**C**) TCP scaffold incorporated by newly formed cancellous bone after 24 weeks. (**D-F**) EDX analysis of histological thin sections (×500 magnification) with (**D**) Mg225d, (**E**) Mg225p and (**F**) TCP particles incorporated by newly formed cancellous bone after 24 weeks. Scale bar = 50 µm.
